# Busulfan systemic exposure and its relationship with efficacy and safety in hematopoietic stem cell transplantation in children: a meta-analysis

**DOI:** 10.1186/s12887-020-02028-6

**Published:** 2020-04-20

**Authors:** Xinying Feng, Yunjiao Wu, Jingru Zhang, Jiapeng Li, Guanghua Zhu, Duanfang Fan, Changqing Yang, Libo Zhao

**Affiliations:** 1 Clinical Research Center, Beijing Children’s Hospital, Capital University of Medical Sciences, Beijing, 100045 China; 2 Department of Hematology and Oncology, Beijing Children’s Hospital, Capital University of Medical Sciences, Beijing, 100045 China; 3 School of Basic Medicine and Clinical Pharmacy, China Pharmaceutical University, Nanjing, 211198 China; 4 Department of Clinical Pharmacy, University of Michigan, Ann Arbor, MI 48109 USA

**Keywords:** Busulfan, Area under the concentration-time curve, Efficacy, Veno-occlusive disease, Meta-analysis

## Abstract

**Background:**

Busulfan (Bu) is a key component of several conditioning regimens used before hematopoietic stem cell transplantation (HSCT). However, the optimum systemic exposure (expressed as the area under the concentration-time curve [AUC]) of Bu for clinical outcome in children is controversial.

**Methods:**

Research on pertinent literature was carried out at PubMed, EMBASE, Web of science, the Cochrane Library and ClinicalTrials.gov. Observational studies were included, which compared clinical outcomes above and below the area under the concentration-time curve (AUC) cut-off value, which we set as 800, 900, 1000, 1125, 1350, and 1500 μM × min. The primary efficacy outcome was notable in the rate of graft failure. In the safety outcomes, incidents of veno-occlusive disease (VOD) were recorded, as well as other adverse events.

**Results:**

Thirteen studies involving 548 pediatric patients (aged 0.3–18 years) were included. Pooled results showed that, compared with the mean Bu AUC (i.e., the average value of AUC measured multiple times for each patient) of > 900 μM × min, the mean AUC value of < 900 μM × min significantly increased the incidence of graft failure (RR = 3.666, 95% CI: 1.419, 9.467). The incidence of VOD was significantly decreased with the mean AUC < 1350 μM × min (RR = 0.370, 95% CI: 0.205–0.666) and < 1500 μM × min (RR = 0.409, 95% CI: 0182–0.920).

**Conclusions:**

In children, Bu mean AUC above the cut-off value of 900 μM × min (after every 6-h dosing) was associated with decreased rates of graft failure, while the cut-off value of 1350 μM × min were associated with increased risk of VOD, particularly for the patients without VOD prophylaxis therapy. Further well-designed prospective and multi centric randomized controlled trials with larger sample size are necessary before putting our result into clinical practices.

## Background

Hematopoietic stem cell transplantation (HSCT) is widely used for the treatment of various malignancies and inherited disorders diseases. High-dose busulfan (Bu) as an alternative to total body irradiation in many pre-transplant conditioning regimens used in clinics today [1]. Although effective, Bu has a relatively narrow therapeutic index, low drug exposure is associated with increased risk of graft failure and disease relapse in transplant recipients [2–4], whereas high drug exposure is associated with increased frequency of hepatic complications, especially veno-occlusive disease (VOD) [5–7]. To improve treatment outcomes of Bu, therapeutic drug monitoring (TDM) and dose adjustment, following the first dose, has highly recommended regardless of the dosing guideline was used [8]. The area under the drug plasma concentration time curve (AUC) or its counterpart, the concentration at steady state (C_SS_) (the AUC divided by dose frequency) best describes the relationship between the pharmacokinetic (PK) and pharmacodynamic (PD) properties of Bu [9].

To our knowledge, there is no conclusive evidence on the relationship between optimum exposure range of Bu and its effectiveness or toxicity in children. The guidelines from the European Medicines Agency (EMA) recommended a target Bu AUC in children of 900 to 1500 μM × min [10]. The FDA labeling recommended a target intravenous (IV) Bu AUC 900 to 1350 ± 5% μM × min after 6 h dosing [8]. The European Society for Blood and Marrow Transplantation (EBMT) guidelines recommend a total AUC after 16 doses of 90 mg × h/L (an equivalent of 1370 μM × min after every 6 h dosage) for myeloablative exposure, without strict distinction between children and adults [11]. Numerous observational studies have recommended target Bu exposure ranges at different cut-off values, including 900 [2, 12–17], 1000 [18], 1225 [11], 1350 [15–17], 1500 [14] and 1575 [11] μM × min for every 6-h dosage. On the contrary, some observational studies found no statistically significant differences in transplant-related toxicity (TRT) or graft failure rate between different Bu AUC [19–21].

Evidence for optimum Bu exposure range described in these studies has obvious limitations. Frist, most of the observational studies that contributed to the aforementioned guidelines had too small a sample size and had no clear inclusion/ exclusion criteria. What’s more, these studies failed to identify different patient groups of adults or children. In light of these uncertainties, we conducted this systematic review and meta-analysis to evaluate the relationship between the reported Bu AUC and clinical outcomes in children undergoing HSCT.

## Methods

### Search strategy

This meta-analysis is reported in accordance with the Cochrane Handbook for Systematic Reviews and the Meta-analysis of Observational Studies in Epidemiology guidelines [22]. Studies were accessed from the PubMed, EMBASE, Web of science, the Cochrane Library and ClinicalTrials.gov. Search terms included “busulfan” in combination with “area under the curve”, “AUC”, “pharmacokinetics*” and “concentration”. Reference lists of retrieved articles and related reviews were also examined, with no language or date restrictions.

### Study selection

Two authors (X.Y.F and Y.J.W) independently applied the inclusion criteria to all identified and retrieved articles, if the two authors could not reach a consensus, a third reviewer (J.R.Z) was brought in to resolve the disagreement. We included studies when: (i) it was an observational study; (ii) Bu was administered 4 times daily for 4 days (16 doses), either orally or by an IV infusion route during the conditioning regimen before HSCT; (iii) TDM was performed; (iv) AUC were reported for included patients; (v) Rate of graft failure and Bu-related adverse events at both below and above the cut-off value of the AUC were reported for included patients, or sufficient data to estimate these was provided; and (vi) sample size was ≥10 patients. The exclusion criteria were as follows: (i) the object of the study was older than 18; (ii) Data came from simulated patients or pharmacokinetic models rather than real patients and; (iii) Clinical data were not presented by Bu AUC strata.

### Cut-off value establishment

According to the cut-off values of target Bu AUC ranges recommended by guidelines from EMA [10], EBMT [11] and the observational studies that we mentioned above [2, 14–17, 20, 23–27] The stepwise cut-off values as 800, 900, 1000, 1225, 1350, and 1500 μM × min was established.

### Data extraction and quality assessment

The primary efficacy outcomes were graft failure (defined as non-engraftment or rejection). The major safety outcomes were VOD incidence and other adverse events. High-risk ratio (RR) denoted a high rate of graft failure, VOD or other adverse events.

Data abstraction was conducted independently by the same two authors (X.Y.F and Y.J.W), and any discrepancy between the investigators was resolved by a third investigator (J.R.Z). The following data were collected and organized from chosen studies: the author’s name, year of publication, study design, number of patients included, methods for measuring Bu concentration, type of AUC (initial, mean or final), cut-off value of Bu AUC, and pre-specified study outcomes of efficacy and safety. Where the study already included the cut-off value, we considered patient groups treated with Bu at an AUC below the pre-defined cut-off value as the treatment group, and those above the pre-defined cut-off value as the control. Where individual patient data were available, we extracted the number of events used all our pre-defined cut-off values to divide patients into two groups in the same way. When the AUC was measured multiple times for each patient, we extracted the first dose AUC (i.e., AUC calculated from 0 h to 6 h after Bu administration) and the mean AUC (i.e., the average value of AUC measured multiple times for each patient). When neither first dose nor mean was available, we used the reported AUC for that patient in the article. When necessary, we contacted the article’s corresponding author by email for the required information.

The quality of the included studies was independently assessed by two reviewers (X.Y.F and Y.J.W) according to the Newcastle–Ottawa Scale with a maximum score of 9 [28]. This tool consists of three major sections concerning the methodological quality: the representative, comparability and outcome of each included study. Any disagreements that arose between the reviewers were resolved through discussion. A third reviewer (J.R.Z) was available to settle disputes.

### Statistical analysis

Data analysis was performed using Open Meta-Analyst software (Tufts Medical Center, Boston, MA, USA). To assess variations between studies in addition to sampling error within these, the *I*^*2*^ statistic was used to assess for heterogeneity across the included studies. An *I*^*2*^ value > 50% suggests substantial heterogeneity between studies. The DerSimonian-Laird was used to calculate RR and 95% confidence interval (CI) for each study. The 95% CI of outcome among distinct groups did not overlap, showing that outcomes were statistically significant. A *P* value < 0.05 was considered statistically significant.

To explore the heterogeneity among different studies, subgroup analysis was performed when more than two studies were included in the analysis of each cut-off level. For the efficacy outcome, studies were stratified by orally or an IV infusion route during the conditioning regimen before HSCT. For the safety outcome, studies were stratified by: i) studies reporting presence or absence of VOD prophylaxis therapy. ii) Orally or an IV infusion route during the conditioning regimen before HSCT. The robustness of our meta-analysis was assessed using leave-one-out approach. We isolated each study and evaluated its effect on the summary estimates and heterogeneity of the main analysis, reporting the results for sensitivity analysis when the conclusions differed.

## Results

### Search strategy and selection criteria

**Fig. 1:**
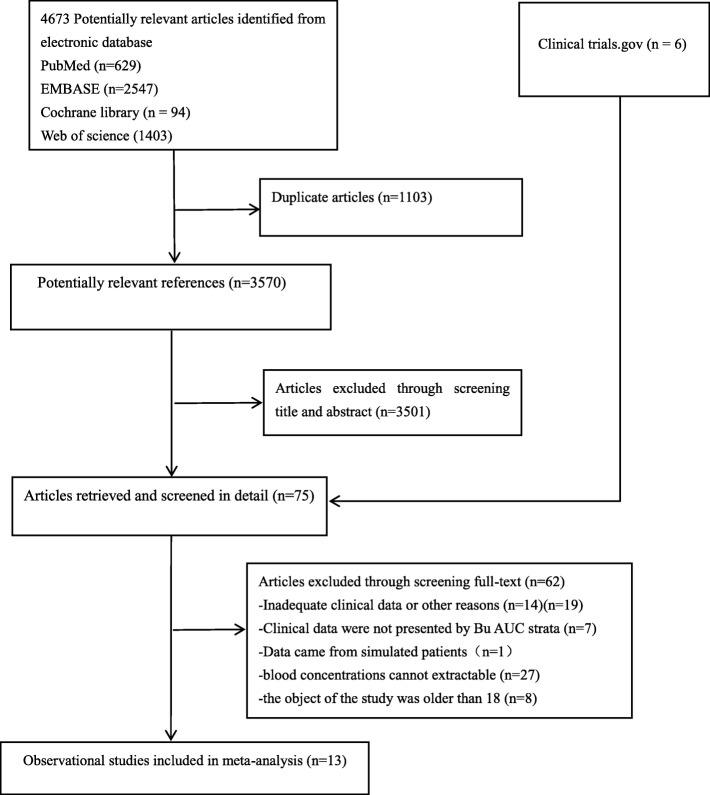
Flow chart of study selection process

A total of 4673 articles were initially identified. Of the 3570 articles remaining after excluding duplicate publications, 3501 were excluded after screening the title and abstract because they were not relevant. An additional 62 articles were excluded during the full-text review owing to data proceeding from simulated patients, the subjects of the study being age over 18, insufficient data on clinical outcomes, clinical data not having been presented by Bu AUC strata or Bu not having been administered 4 times daily for 4 days, among other reasons. Consequently, a total of 13 studies involving 548 patients met the inclusion criteria and, accordingly, were included for meta-analysis [2, 13–17, 20, 23–27, 29]. The literature selection process is summarized in Fig. 1.

### Study characteristics

A summary of descriptions of included studies is reported in Table 1, the studies were published between 1996 and 2017. Nine [13–17, 23, 24, 26, 29] were prospective studies and four [2, 20, 25, 27] were retrospective studies. Six studies were conducted in Europe [14, 16, 20, 24, 25, 27], six studies were in United States [2, 13, 15, 17, 26, 29] and one [23] was in Japan. Bu concentrations were measured by high-performance liquid chromatography by means of ultraviolet detection [23, 29], while the remainder [2, 13–17, 20, 24–27] were measured by gas chromatography with mass spectrometry detection.

**Table 1:** Characteristics of included studies

Reference	Country	Study design	Age (y)^a^	Diagnosis	Dosing	Sampling and analysis	^b^Follow-up (months)
Faraci (2017) [20]	Italy	retrospective	2.9 (1.56–9.9)	NR	iv 0.8–1.2 mg/kg (q6h*4d); po 16 mg/kg or 480 mg/m^2^(q6h*4d)	0,1, 2, 4, and 6 h after the start of infusion/HPLC-UV	48.8 (0.4–139)
Okamoto (2014) [23]	Japan	prospective	6 (0.5–17)	AML (10); ALL (4); CML (2); JMML (5); Others (4)	iv 0.8–1.2 mg/kg (q6h*4d)	1, 2, 2.25, 2.5, 3, and 6 h after the start of infusion/GC-MSD 1,2, 2.5, and 6 h after dose 9; 0, 2.5and 6 h after dose 13/GC-MS	≥3.33
Maheshwari (2013) [13]	USA	prospective	6.2 (1.2–15.5)	SCD	iv1.0 mg/kg or 0.8 mg/kg (q6h*4d)	2, 2.25,2.5,3, 4, 5 and 6 h after the start of infusion/GC-MS	36 (14.4–72)
Veal (2012) [24]	UK	prospective	mean3.6	NB	po 1.45 or 1.55 mg/kg (q6h*4d) iv 0.8–1.2 mg/kg (q6h*4d)	1, 2.25, 2.5, 3 and 6 h after the start of infusion for doses 1 and 9; 0, 2.5, 6 h after the start of infusion for dose 13/GC-MS	≥60
^c^Michel (2011) [14]	France	prospective	–	AML (17); SCD (7); CML (3); NB (27); others (10)	iv 0.8–1.2 mg/kg (q6h*4d)	NR/GC-MS	–
^d^Wall (2009) [15]	USA	prospective	–	AML (8); JMML (2); MDS (2); β-thalassemia (3); Others (9)	iv1.0 mg/kg or 0.8 mg/kg (q6h*4d)	2,3,4,5,6 h after the start of infusion for doses 1 and 9;2 and 6 h after the start of infusion for doses 13 /GC-MS	10.2 (2–23.2)
Vassal (2008) [16]	France	prospective	5.6 (0.3–17.2)	NB (24); AML (14); SCD (5); EWS (3); CML (3); Others (6)	iv 0.8–1.2 mg/kg (q6h*4d)	0, 1, 2, 2.25, 2.5, 3 and 6 h after the start of infusion for doses 1 and 9;0, 2.25 and 6 h after the start of infusion for doses13/GC-MS	NR
^e^Bouligand (2003) [25]	France	retrospective	4.4 (1.1–15.7)	malignant solid tumor	po 37.5 mg/m ^2^ (q6h*4d)	0.5, 2 and 6 h after the start of infusion for dose 1; 6 h after the start of infusion for dose 2, 3, 4, 12,13/GC-MS	NR
Reference	Country	Study design	Age (y)^a^	Diagnosis	Dosing	Sampling and analysis	^b^Follow-up (months)
^f^McCune (2003) [2]	USA	retrospective	6 (0.25–16)	AML (19); MDS (7); SCID (5); others (22)	po total dose 11–28 mg/kg (q6h*4d)	0, 1, 2, 3, 4, 6 h after the start of infusion for dose 5 and dose 9/GC-MS	NR
^f^Bolinger (2001) [26]	USA	prospective	(0.6–17.1)	AML (6); CML (5); β-thalassemia (3); AA (4); SCD (4); others (10)	po total dose 10.9–28.9 mg/kg	0.5, 1, 2, 3, 4, 5, 6 h after the start of infusion for dose 1; 0, 0.5, 1, 2, 4, 6 h after the start of infusion for dose 5, 9 and 13/GC-MS	NR
Bolinger (2000) [17]	USA	prospective	(0.6–18)	β-thalassemia (10); AML (9); others (13)	po total dose 14–20 mg/kg	0, 0.5, 1, 2, 3, 4, 5 and 6 h after the start of infusion for dose 1 and dose 13/GC-MS	NR
Tran (2000) [29]	USA	prospective	7.6 (0.8–18)	ALL (13); AML (7); MDS (3); CML (1); NHL (1)	po 40 mg/m^2^	0, 0.5, 1, 2, 4, 6 h after the start of infusion for dose 1, dose 5 and dose 9/HPLC-UV	32 (11–52)
VASSAL (1996) [27]	France	retrospective	5.9 (1–15)	NB (28); Brain tumors (13); NHL (5); others (11)	po 1 mg/kg or 30–37.5 mg/m^2^	20 min, 40 min as well as 1, 1.5, 2, 3, 4, and 6 h after the start of infusion for dose 1, dose 5 and dose 9/GC-MS	NR

*NR* Not reported, *GC-MS* Gas chromatography with mass spectrometry detection, *IV* Intravenous, *HPLV-UV* High-performance liquid chromatography (HPLC) with the ultraviolet (UV) detection, *AA* Aplastic anemia, *NB* Neuroblastoma, *AML* Acute myeloid leukemia, *ALL* Acute lymphocytic leukemia, *MDS* Myelodysplastic syndrome, *NHL* Non-Hodgkin’s lymphoma, *SCD* Sickle cell disease, *SCID* Severe combined immunodeficiency syndrome, *EWS* Ewing’s sarcoma, *JMML* Juvenile myelomonocytic leukemia; ^a^ age was represented as median (range) or mean ± SD; ^b^Follow-up (moths) was represented as median (interquartile range); ^c^31 patients in the autologous group (aged 0.7 to 14.9 years; median, 4 year), follow up with (49.1 to 56.9 months; median, 52.3 months) and 36 in allogeneic group, (aged 0.3 to 17.2 years old; median, 7.5 years).follow up with (45.5 to 52.8 months; median, 48.5 months);^d^ 13 patients in the ≤4 years group, (aged 0.5 to 3.8 years; median, 1.6 year) and 11 patients in the> 4 years group, (aged 4.5 to 16.7; midian 10.7 years old); ^d^ 13 patients in the ≤4 years group, (aged 0.5 to 3.8 years; median, 1.6 year) and 11 patients in the> 4 years group, (aged 4.5 to 16.7; midian 10.7 years old); ^e^ Bu with MEL group had received more prior chemotherapy courses were not considered for this article; ^f^ 31 patients were accessible for efficacy (one patient older than 18 was not included)

### Evaluation of efficacy

Table 2 displays a summary of outcomes for each study. Table 3 display summaries of meta-analysis for efficacy, Forest plots are shown in Fig. 2. Raw data were shown in Supplementary data (Table S1 and Figures S1–S12).

**Table 2:** Outcomes and results of included studies

Reference	Type of AUC	Cut-off value	Reported outcome	Definition of graft failure or rejection	Definition of VOD
Faraci [20]	Initial	900	Graft failure	NR	Mcdonald criteria [30]
Okamoto [23]	Initial	800; 900; 1000; 1225; 1350; 1500	Graft failure; VOD	Failure to reach ANC > 0.5*10^9^/L by day 28 after transplantation	Mcdonald criteria [30]
maheshwari [13]	Initial and mean	1350; 1500	VOD	NR	McDonald criteria [31]
veal [24]	Mean	1350;1500	Hepatic toxicity or VOD	NR	Bearman criteria [32]
Michel [14]	Mean	900;1350;1500	VOD	NR	McDonald criteria [33]
Wall [15]	Initial, mean and Final	800; 900; 1000; 1225; 1350; 1500	Graft failure, VOD	Failure to reach ANC > 0.5*10^9^/L at any time after transplantation	Jones criteria [34]
vassal [16]	Mean	900;1350;1500	Graft failure; VOD	Failure to reach ANC > 0.5 *10^9^/L for three consecutive days by day 100 after transplantation	Jones criteria [34]
Bouligand [25]	Final	1350;1500	VOD	NR	McDonald criteria [33]
McCune [2]	Mean	900;1350	Graft failure; TRT	Failure to reach ANC > 0.5 *10 ^9^/L	Bearman criteria [32]
Bolinger [26]	Mean	800; 900; 1000; 1225;	Graft failure	No evidence of donor cells or initial evidence of donor engraftment followed by full autologous recovery	Bearman criteria [32]
Bolinger [17]	Initial and mean	800; 900; 1000; 1225;	Graft failure	No evidence of donor cells or initial evidence of donor engraftment followed by full autologous recovery	Bearman criteria [32]
Tran [29]	Mean	1350;1500	VOD	NR	Bearman criteria [32]
VASSAL [27]	Initial	1350;1500	VOD	NR	McDonald criteria [33]

*NR* Not reported, *VOD* Veno-occlusive disease, *TRT* Transplant-related toxicity

**Table 3:** Summary of meta-analyses for the incidence of graft failure

Type of AUC	Cut-off value (μM*min/L)	RR (95% CI)	Number of studies	Number of participants in treatment group	Number of participants in control group	*I*^*2*^%
AUC first dose	< 800 verse ≥800	2.664 (0.857, 8.282)	4	24	67	0
	< 900 verse ≥900	2.208 (0.686, 7.107)	5	73	100	0
	< 1000verse ≥1000	1.544 (0.315, 7.561)	4	48	43	0
	<1225verse ≥1225	1.007 (0.222, 4.578)	4	66	25	0
AUC mean	< 800 verse ≥800	5.296 (1.389, 20.191)	3	22	78	0
	< 900 verse ≥900	3.666 (1.419, 9.467)	7	59	216	0
	<1000verse ≥1000	1.245 (0.267, 5.809)	4	62	38	0
	<1225verse ≥1225	0.559 (0.125, 2.505)	4	78	22	0

*CI* Confidence interval

**Fig. 2:**
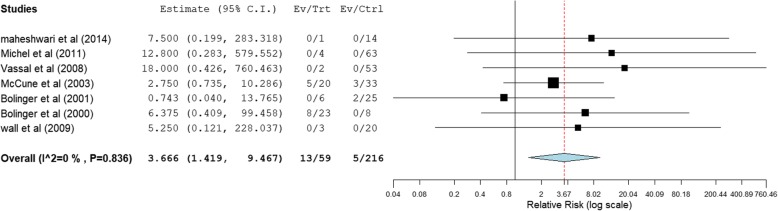
Meta-analysis for rate of graft failure (mean AUC of < 900 μM × min comparison with ≥900 μM × min, RR <1 favors ≥900 μM × min)

Our meta-analysis demonstrated that there were no significant first dose AUC cut-off values for efficacy. We found the cut-off level (AUC mean) of < 900 μM × min to be significantly associated with higher incidence of graft failure (RR = 3.666, 95% CI: 1.419, 9.467).

Subgroup analyses showed that the incidence of graft failure significantly decreased above a cut-off level with mean AUC 900 μM × min in the subgroup of administration by an IV infusion route alone (RR = 9.718; 95% CI: 1.499–62.989), There were no significant differences at other cut-off levels (Table 4).

**Table 4:** Summary of subgroup analysis for the incidence of graft failure

Subgroup		Cut-off value (μM*min/L)	RR (95% CI)	Number of studies	Number of participants in treatment group	Number of participants in control group	*I*^*2*^%
Administration route	IV Bu	≤800 versus> 800	11.282 (0.930, 136.897)	2	2	36	0
		≤900 versus> 900	9.718 (1.499, 62.989)	4	10	150	0
		≤1000 versus> 1000	0.418 (0.030, 5.850)	2	23	15	0
		≤1225 versus> 1225	0.139 (0.011, 1.729)	2	32	6	0
	Oral Bu	≤800 versus> 800	3.904 (0.800,19.055)	2	20	42	0
		≤900 versus> 900	2.613 (0.869,7.860)	3	49	66	0
		≤1000 versus> 1000	2.189 (0.328,14.587)	2	39	23	0
		≤1225 versus> 1225	1.197 (0.186,7.720)	2	46	16	0

*CI* Confidence interval, *NA* Not applicable, *IV* Intravenous

Sensitivity analysis on each study’s effect on the summary estimates for efficacy was shown in Supplementary data (Table S3), which illustrated that our results were not driven by any single study, as the RRs remained stable.

### Evaluation of safety

A summary of primary and subgroup analysis for safety are shown in Table 5 and Table 6. Forest plots are shown in Fig. 3 and Fig. 4. Raw data were shown in Supplementary data (Table S2 and Figures S13-20).

**Table 5:** Summary of meta-analyses for the incidence of VOD

Type of AUC	Cut-off value (μM*min/L)	RR (95% CI)	Number of studies	Number of participants in treatment group	Number of participants in control group	*I*^*2*^%
AUC first dose	≤1350 versus>1350	0.562 (0.126,2.496)	3	51	23	26.96%
	≤1500 versus>1500	0.761 (0.435,1.333)	4	87	44	0
AUC mean	≤1350 versus>1350	0.370 (0.205,0.666)	7	207	61	0
	≤1500 versus>1500	0.409 (0.182,0.920)	5	163	28	0

*CI* Confidence interval

**Table 6:** Summary of subgroup analysis for incidence of VOD

Sub group		Cut-off value (μM*min/L)	RR (95% CI)	Number of studies	Number of participants in treatment group	Number of participants in control group	*I*^*2*^*%*
Administration route	IV Bu alone	≤1350 versus> 1350	0.378 (0.158,0.906)	3	106	30	0
		≤1500 versus> 1500	0.485 (0.171,1.377)	3	129	17	0
	IV Bu + oral Bu/oral Bu	≤1350 versus> 1350	0.363 (0.163, 0.805)	4	101	31	0
		≤1500 versus> 1500	0.316 (0.087,1.145)	2	34	11	0
VOD prophylaxis	Yes	≤1350 versus> 1350	0.476 (0.120, 1.885)	1	42	15	NA
		≤1500 versus> 1500	0.491 (0.109, 2.216)	1	56	11	NA
	No	≤1350 versus> 1350	0.349 (0.182, 0.670)	6	165	46	0
		≤1500 versus> 1500	0.380 (0.145, 0.994)	4	107	17	0

*CI* Confidence interval, *NA* Not applicable, *IV* Intravenous

**Fig. 3:**
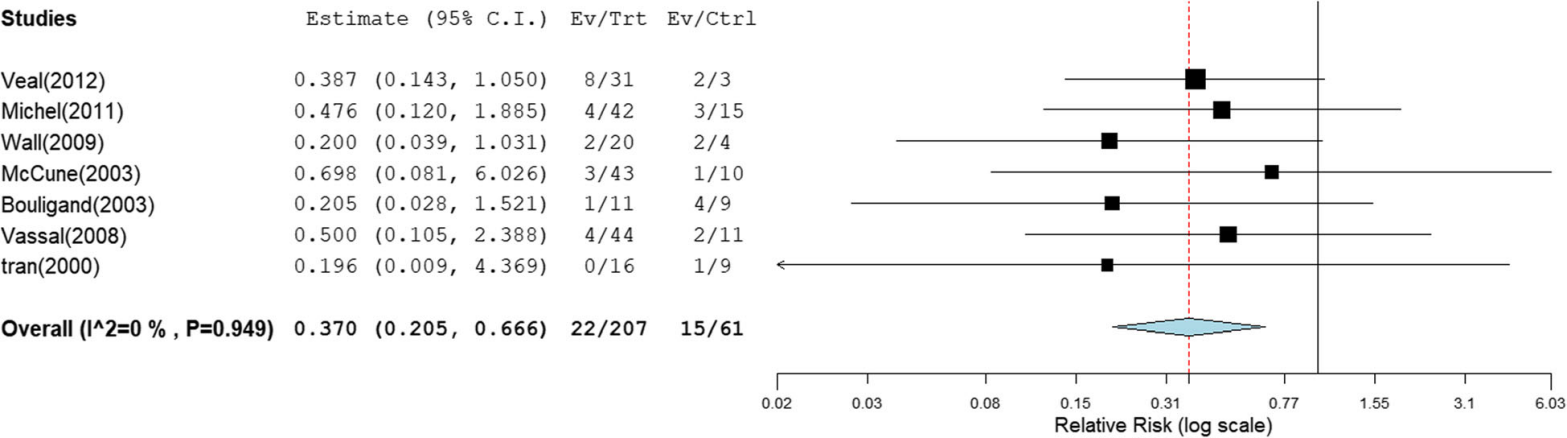
Meta-analysis for incidence of VOD (mean AUC of < 1350 μM × min comparison with ≥1350 μM × min, RR < 1 favors ≥1350 μM × min)

**Fig. 4:**
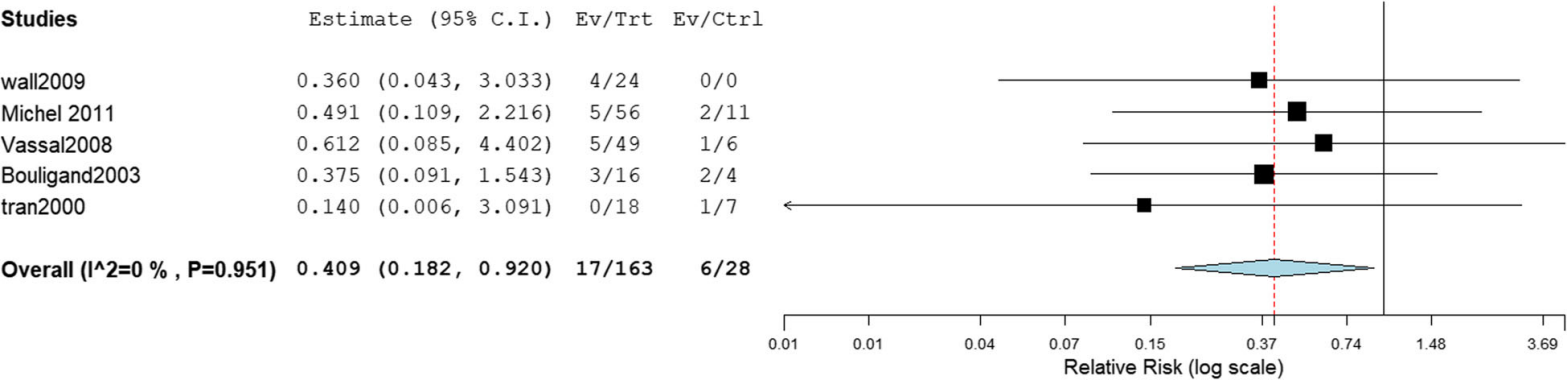
Meta-analysis for incidence of VOD (mean AUC of < 1500 μM × min comparison with ≥1500 μM × min, RR < 1 favors ≥1500 μM × min)

The definitions of VOD varied across the 10 studies (Table 2), the incidence of VOD ranged from 4.8% [2, 13–17, 20, 24–27] to 40% [27]. On average, VOD occurred between 1 and 29 days after HSCT. Our meta-analysis demonstrated a significantly lower incidence of VOD with mean AUC below cut-off levels of 1350 μM × min (RR = 0.370, 95% CI: 0.205–0.666) and 1500 μM × min (RR = 0.409, 95% CI: 0182–0.920). In terms of the relationship between first dose AUC and clinical outcomes, our meta-analysis demonstrated there were no significant differences at all cut-off values for VOD.

Subgroup analyses showed that the rate of VOD significantly decreased below a cut-off level with mean AUC 1350 μM × min in the subgroup of without VOD prophylaxis therapy (RR = 0.349; 95% CI: 0.182–0.670), administration by an IV infusion route alone (RR = 0.378; 95% CI: 0.158–0.906) or not (either administration by an IV infusion route or by oral) (RR = 0.363; 95% CI: 0.163–0.805). There were no significant differences at other cut-off levels.

For others toxic effects, the relationship of Bu AUC with graft versus-host disease (GVHD) was not found, although two studies [35, 36] reported a higher incidence of GVHD when Bu/cyclophosphamide was combined with melphalan. Regarding neurotoxicity, as benzodiazepine or phenytoin was routinely given for seizure prophylaxis, the incidence of neurotoxicity was relatively low. We could not pool the data to perform a meta-analysis. Therefore, an association between AUC and other toxic effects could not be evaluated.

On each study’s effect on the summary estimates showed that exclusion of studies by Wallet al [15], Bouligand et al. [25] and Tran et al. [29] resulted in an insignificant difference at a cut-off level of 1500 μM × min Raw data were shown in Supplementary data (Table S4).

### Quality assessment

The quality assessment of the included studies is presented in Supplementary Table S5. Overall, the subjects included were representative, and ascertainment of exposure was confirmed by secure record, six studies were comparable on basis of main factors [2, 14–16, 24, 25], and seven studies were comparable on two or more factors [13, 17, 20, 23, 26, 27, 29]. Outcome assessment was based on pharmacy and medical records, the follow-up period was sufficient for outcomes to occur, and adequacy of follow-up of cohorts. According to the NOS tool, the quality assessment showed that two studies [17, 26] were scored 6 stars, four studies 7 stars [20, 25, 27, 29], three studies [13, 16, 23] 8 stars, and four studies [2, 14, 15, 24] 9 stars. No study was excluded after rating because the study quality was always above 5 stars.

## Discussion

As a bifunctional alkylating agent, Bu is a key component of several conditioning regimens used before HSCT. It has been demonstrated that low plasma Bu exposure is associated with potentially fatal outcomes including graft failure, whereas high exposure is associated with toxicity, such as VOD [3, 5, 7]. Due to the high inter- and intra-patient variability in the PK profile following oral and IV infusion [10], major guidelines support and recommend TDM for Bu to improve transplant outcomes [9, 26, 37], although the exact therapeutic window in children remains inconclusive.

Our meta-analysis revealed that a Bu mean AUC above the value 900 μM × min is associated with lower incidence of graft failure. This lower threshold of exposure is similar to the guideline recommendation [8]. We conducted a subgroup analysis by orally or by an IV infusion route during the conditioning regimen before HSCT, thereby demonstrating that the incidence of graft failure significantly decreased at a cut-off level of > 900 μM × min in subgroup of administration by an IV infusion route. As we know, oral Bu presents a wide inter- and intrapatient variability of plasma exposures, especially in young children, which results in poor clinical outcomes [35]. That might explain why the oral Bu subgroup did not show significance at the 900 μM × min cut-off level. Our sensitivity analysis further validated the cut-off value 900 μM × min for efficacy. In addition, numerous studies [19, 35] have found that the first-dose Bu AUC was significantly lower than the subsequent daily ones and AUC remained unchanged during the following days. However, we cannot identify the relationship between AUC at the first dose and efficacy as there is insufficient data from studies to support this. Thus, the correlation remain inconclusive and further investigation is needed.

Our meta-analysis also demonstrated that a target value of 1350 μM × min is associated with an increased risk of VOD. This conclusion differs from the 900–1500 μM × min threshold that some publications [11, 12, 15] have suggested. This is likely due to the fact that those studies are mainly conducted on adults and their subjects of study are relatively limited. In our subgroup analyses, we stratified studies according to administration route and whether Bu treatment was combined with VOD prophylaxis therapy. In subgroup patients without VOD prophylaxis therapy, a significantly decreased incidence of VOD was detected when Bu AUC was below the cut-off value of 1350 μM × min, which could not be seen in those patients with VOD prophylaxis therapy. Plausible explanations are as follows. First, only high-risk patients (pre-existing liver damage, history of pancreatitis, genetic polymorphisms and mutations) were considered eligible for VOD prophylaxis therapy [38], which may have physiological effects on identifying the relationship between drug exposure and VOD. Secondly, as there are only two studies that include patients with VOD prophylaxis therapy, we regard these subgroup analysis results as likely to be unreliable.

The optimum Bu AUC of 900–1350 μM × min is consistent with some previous research recommendations [15, 39], but differs from a recently multicenter, retrospective cohort analysis reported by Bartelink et al. [11] which showed that, in children and young adults, the optimum Bu AUC is at a cumulative AUC of 78–101 mg × h/L (equivalent to 1225–1575 μM × min after every 6 h dosing). However, there were some discrepancies that should be noted. We enforced a restriction on enrolled patients being less than 18 years of age and to be administered with Bu 4 times a day for 4 days, while in the study by Bartelink et al. [11], patients older than 18 were included and Bu was given once or four times a day. These differences in age and frequency of administration might lead to a different optimum AUC.

Our study has several strengths. First and foremost, it is the first meta-analysis focusing on the relationship of Bu AUC with efficacy and safety in children, providing certain reference to individualized therapy. Secondly, our meta-analysis allowed for comparison of commonly used cut-off levels for efficacy and safety in a single analysis for individual cut-off levels. Finally, our study takes the approaches of AUC estimation (AUC for the first dose or the mean value) among transplant centers into consideration, which allowed us carry out more comprehensive comparisons of Bu AUC, despite the fact that the patients came from different institutions.

We acknowledge the following limitations to our work. First, due to the shortage of available data, a detailed analysis according to different conditioning regimens and underlying disease (malignant or non-malignant disease) was not performed, which may have drug-drug interaction, and physiological effects on identifying the cut-off value of drug exposure (patients with a different disease should be treated as separate populations as they may respond to treatment differently). Moreover, we were unable to include enough data from Asian location, because we only identified one study conducted in Japan [23]. This is a timely reminder that the optimized AUC should be considered with caution when applying the results in Asian location. Finally, the use of observational studies in the meta-analysis implies biases and confounding factors, given that these are inherent in the original studies. As such, there is a clear requirement for further research.

## Conclusion

This meta-analysis demonstrated that Bu mean AUC above the cut-off value of 900 μM × min (after every 6-h dosing), was associated with decreased rates of graft failure, while the cut-off value of 1350 μM × min were associated with increased risk of VOD in children, particularly for the patients without VOD prophylaxis therapy. However, our result is a synthesis of observational studies, which are the relatively low-level evidence, and should be treated carefully. Further well-designed prospective and multi centric randomized controlled trials with larger sample size are necessary before putting our result into clinical practices.

## Supplementary information

**Additional file 1.** Supplementary data.

## Data Availability

Raw data from this review is available in Supplementary data.

## Abbreviations

AUC: Area under the drug plasma concentration time curve
RR: Relative risk
HSCT: Hematopoietic stem cell transplantation
TDM: Therapeutic drug monitoring
VOD: Veno-occlusive disease
NOS: Newcastle–Ottawa Scale
