# Supplementary material for: Busulfan systemic exposure and its relationship with efficacy and safety in hematopoietic stem cell transplantation in children: a meta-analysis
Source: BMC Pediatr. 2020 Apr 20;20:176. doi: 10.1186/s12887-020-02028-6 (PMC7168843; doi:10.1186/s12887-020-02028-6)
Supplement: Supplementary file 1 — Additional file 1. Supplementary data. [file 12887_2020_2028_MOESM1_ESM.doc]

**supplementary data**

**Contents**

**Appendix 1 Busulfan systemic exposure and its relationship with efficacy and safety in hematopoietic stem cell transplantation in children (Protocol).**

**Appendix 2 Raw data for efficacy (Table S1)**

**Appendix 3 Raw data for safety (Table S2)**

**Appendix 4 Sensitivity analysis on each study’s affect on the summary estimates for efficacy (Table S3)**

**Appendix 5 Sensitivity analysis on each study’s affect on the summary estimates for safety (Table S4)**

**Appendix 6 Quality appraisal of included studies (Table S5)**

**Appendix 7 Forest plot for efficacy and safety at each cutoff value (Figure S1-S16)**

**7.1 Forest plot for rate of** **graft failure at each cutoff value (Figure** **S1-S8)**

**7.2 Forest plot for subgroup analysis of rate of graft failure at each cutoff value (Figure S9-S12)**

**7.3** **Forest plot for safety at each cutoff value (Figure S13-S16)**

**7.****4 Forest plot for subgroup analysis of rate of veno-occlusive disease (VOD) at each cutoff value (Figure S17-S20)**

**Appendix 1 Busulfan systemic exposure and its relationship with efficacy and safety in hematopoietic stem cell transplantation in children (Protocol).**

Xinying Feng, Yunjiao Wu, Jingru Zhang, Jiapeng Li, Guanghua Zhu, Duanfang Fan, ChangqingYang, Libo Zhao

**BACKGROUND**

**Description of the condition**

Hematopoietic stem cell transplantation (HSCT) is the standard treatment for various malignant and non-malignant disorders (eg, immunodeficiencies, inherited metabolic diseases, and haemoglobinopathies) (Bartelink 2016).

**Description of the intervention**

Busulfan (Bu) is an alkylating drug routinely used in conditioning regimens before HSCT (Bartelink 2016). Although effective, Bu has a relatively narrow therapeutic index, low drug exposure is associated with increased risk of graft failure and disease relapse in transplant recipients (Grochow 1993; Slattery 1995; McCune 2002;), whereas high drug exposure is associated with increased frequency of hepatic complications, especially veno-occlusive disease (VOD) (Geddes 2008; Copelan 2001). To improve treatment outcomes of Bu, therapeutic drug monitoring (TDM) and dose adjustment, following the first dose, has highly recommended regardless of the dosing guideline was used (Palmer 2016). The area under the drug plasma concentration time curve (AUC) or its counterpart, the concentration at steady state (C_SS_) (the AUC divided by dose frequency) best describes the relationship between the pharmacokinetic (PK) and pharmacodynamic (PD) properties of Bu (Palmer 2016).

**Why it is important to do this review**

There is no conclusive evidence on the relationship between optimum exposure range of Bu and its effectiveness or toxicity in children. The guidelines from the European Medicines Agency (EMA) recommended a target Bu AUC in children of 900 to 1500 µM × min (Nguyen 2004). The FDA labeling recommended a target intravenous (IV) Bu AUC 900 to 1350 ± 5% µM × min after 6 hour dosing. The European Society for Blood and Marrow Transplantation (EBMT) guidelines recommend a total AUC after 16 doses of 90 mg*h/L (an equivalent of 1370 µM × min after every 6 hour dosage) for myeloablative exposure, without strict distinction between child and adult (Bartelink 2016). Numerous observational studies have recommended target Bu exposure ranges at different cut-off values, including 900(McCune 2002), 1000 (Zwaveling 2005), 1225 (Bartelink 2016), 1350 (Bolinger 2000), 1500(Michel 2011) and 1575 (Bartelink 2016) µM × min for every 6-hour dosage. On the contrary, some observational studies found no statistically significant differences in transplant-related toxicity (TRT) or graft failure rate between different Bu AUC (Faraci 2017).

**OBJECTIVES**

To evaluate the relationship between the reported Bu AUC and clinical outcomes in children undergoing HSCT

**METHODS**

**Criteria for considering studies for this review**

**Types of studies**

Observational studies that meet the eligibility criteria will be included in this study. There will be no restrictions regarding the language, but only the studies published as full text will be included.

**Types of participants**

Pediatric patients (aged ≤18 years) affected by malignant or non-malignant diseases that received high-dose Bu before undergoing HSCT will be included. Bu should be administered 4 times daily for 4 days based on the patient's body weight (BW) or body surface area (BSA) at a dose of 0.8-1.2 mg/kg or 30-40 mg/m^2^.

**Types of interventions**

Cut-off value establishment

According to the cut-off values of target Bu AUC ranges recommended by guidelines from EMA(Nguyen 2004), EBMT (Bartelink 2016) and the observational studies, the stepwise cut-off values as 800, 900, 1000, 1225, 1350, and 1500 µM × min are established .

- Treatment group, patient groups that below the pre-defined cut-off value;
- Control group, patient groups that above the pre-defined cut-off value.

**Types of outcome measures**

- Graft failure

The primary efficacy outcome will be the incidence of graft failure, defined as the failure to reach an absolute neutrophil count (ANC) of 0.5 × 10^9^/L by day 28 after transplantation

- Veno-occlusive disease (VOD)

Primary safety outcome will be the incidents of VOD, defined as presence of two or more of the following occurring within 21 days after transplant (in the absence of other causes of liver disease): bilirubin 2 mg/mL, unexpected weight gain 5% of baseline weight or presence of ascites, and hepatomegaly or right upper quadrant tenderness.

If the necessary data are available, we will analyses the following outcomes as secondary outcomes:

- Relapse, recorded as the day of detection of disease recurrence;
- Acute graft-versus-host disease (GVHD), assessed according to consensus criteria. (Przepiorka D 1995);
- Regimen-related toxicity, assessed by the National Cancer Institute Common Toxicity Criteria (CTC) version 2.0, or by Bearman grading (Bearman 1998);
- Event-free survival (EFS), calculated from the time of transplant until death, relapse or graft failure；
- Overall survival (OS), defined as the time from transplantation to death, irrespective of the cause. EFS and OS after HSCT were evaluated for patients with a follow-up of at least 9 months.

**Search methods for identification of studies**

**Electronic searches**

We will search for studies as described in Chapter 6 of the Cochrane Handbook of Systematic reviews of Interventions (Lefebvre 2011). Search terms will include “busulfan” in combination with “area under the curve”, “AUC”, “pharmacokinetics*” and “concentration”. There will be no language and date, limitations for inclusion of records into the register. If we identify any papers in a language not known by the review author team, we will seek help outside of the review author group. We will acknowledge any assistance received in the “Acknowledgements” section of the published review.

We will search the following databases and sources

- Cochrane Central Register of Controlled Trials (CENTRAL) (Cochrane Library; latest issue) in the Cochrane Library;
- MEDLINE, accessed via PubMed;
- Embase Ovid;
- Web of Science;
- ClinicalTrial.gov ([clinicaltrials.gov/](https://clinicaltrials.gov/)).

**Searching other resources**

We will inspect references of all included studies for further relevant studies.

**Data collection and analysis**

We will conduct this meta-analysis according to The Cochrane Handbook for Systematic Reviews (Higgins 2011) and the Meta-analysis of Observational Studies in Epidemiology guidelines (Stroup 2008).

**Selection of studies**

Two review authors (X.Y.F and Y.J.W) will independently screen the results of the search strategies for eligibility for this review by reading the abstracts. In the case of disagreement the full text publication will be obtained. If no consensus can be reached, we will ask a third review author (J.R.Z).

Two review authors (X.Y.F and Y.J.W) will independently screen the results of the search strategies for eligibility for this review by reading the abstracts. We will retrieve full texts of potentially eligible studies for further assessment, and the two authors will independently apply the inclusion criteria to these publications. Disagreements between the two authors with regard to both the screening and full-text assessment will be resolved through discussion and consensus. J.R.Z will serve as the arbitrator to resolve disagreements that X.Y.F and Y.J.W unable to resolve through discussion and consensus.

We will document the process of study selection in a flow chart, as recommended by the Preferred Reporting Items for Systematic Reviews and Meta-Analyses (PRISMA) statement (Moher 2009), showing total numbers of retrieved references and numbers of included and excluded studies.

**Data extraction and management**

Review authors (X.Y.F and Y.J.W) will extract data from all included studies, any discrepancy between the investigators will be resolved by a third investigator (J.R.Z). In addition, to ensure reliability, L.B.Z will independently extract data from a random sample of these studies, comprising 10% of the total. We will attempt to extract data presented only in graphs and figures whenever possible, but will include this information only if two review authors independently obtain the same result. Authors will not review articles they have consulted on, authored, or co-authored. We will extract the following data:

- General information

The author’s name, year of publication, country, language, title, source, duplicate publications;

- Participant characteristics

Age, sex, diagnosis, number of patients included, number of participants in treatment group, number of participants in control group;

- Intervention details

Cut-off value, methods for measuring Bu concentration, type of AUC (initial, mean or final);

- Outcomes

Pre-specified study outcomes of efficacy and safety, [follow-up](../../../../C:/Users/feng/AppData/Local/youdao/dict/Application/8.8.0.0/resultui/html/index.html#/javascript:;) [time](../../../../C:/Users/feng/AppData/Local/youdao/dict/Application/8.8.0.0/resultui/html/index.html#/javascript:;).

**Dealing with missing data**

We will personally contact the lead authors of the trials for further information, if required.

**Assessment of risk of bias in included studies**

The quality of the included studies will be independently assessed by two reviewers (X.Y.F and Y.J.W) according to the Newcastle–Ottawa Scale with a maximum score of 9; any disagreements that arose between the reviewers will be resolved through discussion. A third reviewer (J.R.Z) will be available to settle disputes. We will assess the risk of bias according to the following domains.

1. Indicates exposed cohort truly representative;

2. Non-exposed cohort drawn from a same source;

3. Ascertainment of exposure from a secure record;

4. Outcome of interest not present at start of study;

5A. Cohorts comparable on basis of main factor;

5B.Cohorts comparable on other factors;

6. Assessment of outcome of record linkage or independent blind assessment;

7. Follow-up long enough for outcomes to occur;

8. Complete accounting for cohorts.

**Assessment of reporting biases**

We will use Begg’s test and Egger’s weighted regression statistics to assess reporting bias if more than 10 studies would be included in the analysis of each cut-off level (Deeks 2011).

**Data synthesis**

Meta-analysis and assessment of publication bias will be performed using the Open Meta-Analyst software (Tufts Medical Center, Boston, MA, USA) and Stata version 12.0 (StataCorp LP). To assess variations between studies in addition to sampling error within these, the I^2^ statistic was used to assess for heterogeneity across the included studies. An I^2^value >50% suggests substantial heterogeneity between studies. For dichotomous (or binary) data, risk ratio (RR) with 95% confidence intervals will be used. The DerSimonian–Laird will be used to calculate RR and 95% confidence interval (CI) for each study. A *P* value <0.05 will be considered statistically significant.

**Subgroup analysis and investigation of heterogeneity**

If the necessary data are available, we will perform the following subgroup analyses.

- Studies reporting presence or absence of VOD prophylaxis therapy;
- Different types of Bu administration (oral tablets versus intravenous infusion);
- Study location.

**Sensitivity analysis**

The robustness of our meta-analysis will be assessed using leave-one-out approach. We will isolate each study and evaluate its effect on the summary estimates and heterogeneity of the main analysis. We will carry out sensitivity analyses for primary outcomes only.

**REFERENCES**

**Additional references**

Bartelink 2016

Bartelink IH, Lalmohamed A, van Reij EM, Dvorak CC, Savic RM, Zwaveling J, Bredius RG, Egberts AC, Bierings M, Kletzel M et al: Association of Bu exposure with survival and toxicity after haemopoietic cell transplantation in children and young adults: a multicentre, retrospective cohort analysis. Lancet Haematol 2016; 3(11):e526-e536.

Palmer 2016

Palmer J, Mccune JS, Perales MA, Marks D, Carpenter PA: Personalizing Busulfan-Based Conditioning: Considerations From the American Society for Blood and Marrow Transplantation Practice Guidelines Committee. Biology of Blood & Marrow Transplantation Journal of the American Society for Blood & Marrow Transplantation 2016; 22(11):1915-1925

Slattery 1995

Slattery JT, Sanders JE, Buckner CD, Schaffer RL, Lambert KW, Langer FP, Anasetti C, Bensinger WI, Fisher LD, Appelbaum FR et al: Graft-rejection and toxicity following bone marrow transplantation in relation to busulfan pharmacokinetics. Bone Marrow Transplant 1995; 16(1):31-42.

Grochow 1993

Grochow LB: Busulfan disposition: the role of therapeutic monitoring in bone marrow transplantation induction regimens. SEMIN ONCOL 1993; 20(4):18-25.

Geddes 2008

Geddes M, Kangarloo SB, Naveed F, Quinlan D, Chaudhry MA, Stewart D, Savoie ML, Bahlis NJ, Brown C, Storek J: High busulfan exposure is associated with worse outcomes in a daily i.v. busulfan and fludarabine allogeneic transplant regimen. Biology of Blood & Marrow Transplantation Journal of the American Society for Blood & Marrow Transplantation 2008; 14(2):220-228.

Zwaveling 2005

Zwaveling J, Bredius RG, Cremers SC, Ball LM, Lankester AC, Teepe-Twiss IM, Egeler RM, Den HJ, Vossen JM: Intravenous busulfan in children prior to stem cell transplantation: study of pharmacokinetics in association with early clinical outcome and toxicity. BONE MARROW TRANSPL 2005; 35(1):17-23.

Bolinger 2000

Bolinger AM, Zangwill AB, Slattery JT, Glidden D, Desantes K, Heyn L, Risler LJ, Bostrom B, Cowan MJ: An evaluation of engraftment, toxicity and busulfan concentration in children receiving bone marrow transplantation for leukemia or genetic disease. BONE MARROW TRANSPL 2000; 25(9):925-930.

Michel 2011

Michel G, Valteaucouanet D, Gentet JC, Esperou H, Socié G, Méchinaud F, Doz F, Neven B, Bertrand Y, Galambrun C: Weight-based strategy of dose administration in children using intravenous busulfan: clinical and pharmacokinetic results. PEDIATR BLOOD CANCER 2011; 58(1):90-97.

Faraci 2017

Faraci M, Tinelli C, Lanino E, Giardino S, Leoni M, Ferretti M, Castagnola E, Broglia M, Silvestri AD, Martino DD: Monitoring of Busulphan Concentrations in Children Undergone Hematopoietic Stem Cell Transplantation: Unicentric Experience over 10 years. European Journal of Drug Metabolism & Pharmacokinetics 2017; 43(Suppl 2):1-9.

Copelan 2001

Copelan EA, Bechtel TP, Avalos BR, Elder PJ, Ezzone SA, Scholl MD, Penza SL: Busulfan levels are influenced by prior treatment and are associated with hepatic veno-occlusive disease and early mortality but not with delayed complications following marrow transplantation. Bone Marrow Transpl 2001; 27(11):1121.

McCune 2002

McCune JS, Gooley T, Gibbs JP, Sanders JE, Petersdorf EW, Appelbaum FR, Anasetti C, Risler L, Sultan D, Slattery JT: Busulfan concentration and graft rejection in pediatric patients undergoing hematopoietic stem cell transplantation. Bone Marrow Transplant 2002; 30(3):167-173.

Higgins 2011

Higgins JP, Green S, editor(s). Cochrane Handbookfor Systematic Reviews of Interventions Version 5.1.0(updated March 2011). The Cochrane Collaboration, 2011. Available from handbook.cochrane.org. 5.1. The Cochrane Collaboration, (accessed 15th March 2018).

Moher 2009

Moher D, Liberati A, Tetzlaff J, Altman DG. The PRISMA Group. Preferred reporting items for systematic reviews and meta-analyses: the PRISMA statement. Journal of Clinical Epidemiology 2009;62(10):1006–12.

Stroup 2008

Stroup DF, Berlin JA, Morton SC, Olkin I, Williamson GD, Rennie D, Moher D, Becker BJ, Sipe TA, Thacker SB: Meta-analysis of observational studies in epidemiology: a proposal for reporting. Meta-analysis Of Observational Studies in Epidemiology (MOOSE) group. 2008.

Lefebvre 2011

Lefebvre C, Manheimer E, Glanville J. Chapter 6: Searching forstudies. In: Higgins JP, Green S, editor(s). Cochrane Handbook for Systematic Reviews of Interventions Version 5.1.0 (updatedMarch 2011). The Cochrane Collaboration, 2011.Available fromhandbook.cochrane.org.

Przepiorka D 1995

Przepiorka D, Weisdorf D, Martin P, Klingemann HG, Beatty P, Hows J, Thomas ED. Consensus conference on acute GVHD. Bone Marrow Transplant 1995; 15:825–828.

Nguyen 2004

Nguyen L, Fuller D, Lennon S, Leger F, Puozzo C: I.V. busulfan in pediatrics: a novel dosing to improve safety/efficacy for hematopoietic progenitor cell transplantation recipients. BONE MARROW TRANSPL 2004; 33(10):979.

Bartelink 2016

Bartelink IH, Lalmohamed A, van Reij EM, Dvorak CC, Savic RM, Zwaveling J, Bredius RG, Egberts AC, Bierings M, Kletzel M et al: Association of busulfan exposure with survival and toxicity after haemopoietic cell transplantation in children and young adults: a multicentre, retrospective cohort analysis. LANCET HAEMATOL 2016; 3(11):e526-e536.

Deeks 2011

Deeks JJ, Higgins JPT, Altman DG. Chapter 9: Analysing data and undertaking meta-analyses. In: Higgins J, Green S (eds). Cochrane Handbook for Systematic Reviews of Interventions Version 5.1.0.(Updated March 2011).The Cochrane Collaboration, 2011. http://www.cochrane-

handbook.org

Bearman 1988

Bearman SI, Appelbaum FR, Buckner CD, Petersen FB, Fisher LD, Clift RA, Thomas ED: Regimen-related toxicity in patients undergoing bone marrow transplantation. Journal of Clinical Oncology Official Journal of the American Society of Clinical Oncology 1988; 6(10):1562-1568.

**CONTRIBUTIONS OF AUTHORS**

LBZ and CQY conceived the idea for this protocol. XYF drafted the protocol. LBZ, CQY, JRZ, YJW, JPL, DFF and GHZ reviewed the protocol and provided critical feedback. LBZ is the guarantor for this review.

**Appendix 2 Raw data for** **efficacy (Table S1)**

**Table S1 Raw data for rate of graft failure with different cutoff value**

| **Reference** | **Type of AUC** | **800** µM × min | | **900** µM × min | | **1000** µM × min | | **1225** µM × min | |
| --- | --- | --- | --- | --- | --- | --- | --- | --- | --- |
|  |  | **≤800** | **＞800** | **≤900** | **＞900** | **<1000** | **＞1000** | **≤1225** | **＞1225** |
| Okamoto 2014 | AUC(Initial) | 0/1 | 2/24 | 0/2 | 2/23 | 0/3 | 2/22 | 1/7 | 1/18 |
| Faraci 2017 | AUC(Initial) | NR | NR | 3/36 | 2/46 | NR | NR | NR | NR |
| Maheshwari 2014 | AUC(Initial) | 0/4 | 0/11 | 0/7 | 0/8 | 0/11 | 0/4 | 0/13 | 0/2 |
| Maheshwari 2014 | AUC(mean) | 0/0 | 0/15 | 0/1 | 0/14 | 0/14 | 0/1 | 0/15 | 0/0 |
| Vassal 2008 | AUC(mean) | NR | NR | 0/2 | 0/53 | NR | NR | NR | NR |
| Michel 2011 | AUC(mean) | NR | NR | 0/4 | 0/63 | NR | NR | NR | NR |
| McCune 2003 | AUC(mean) | NR | NR | 5/20 | 3/33 | NR | NR | NR | NR |
| Bolinger 2001 | AUC(mean) | 0/1 | 2/30 | 0/6 | 2/25 | 1/12 | 1/19 | 1/17 | 1/14 |
| ^a^ Bolinger 2000 | AUC(Initial) | 6/15 | 2/13 | 8/21 | 0/7 | 8/22 | 0/6 | 8/26 | 0/2 |
| Bolinger 2000 | AUC(mean) | 7/19 | 1/12 | 8/23 | 0/8 | 8/27 | 0/4 | 8/29 | 0/2 |
| ^b^Bolinger 2000 | AUC(final) | 5/17 | 1/10 | 6/20 | 0/7 | 6/24 | 0/3 | 6/25 | 0/2 |
| Wall 2009 | AUC(Initial) | 0/4 | 0/19 | 0/7 | 0/16 | 0/12 | 0/11 | 0/20 | 0/3 |
| Wall 2009 | AUC(mean) | 0/2 | 0/21 | 0/3 | 0/20 | 0/9 | 0/14 | 0/17 | 0/6 |
| ^c^Wall 2009 | AUC(final) | 0/0 | 0/22 | 0/1 | 0/21 | 0/6 | 0/16 | 0/14 | 0/8 |

AUCInitial,AUC obtained after the first dose of busulfan; AUC mean, mean value of multiple measurements;AUC final,measurements after dose adjustment.

NR=not reported.

^a^ Bolinger 2000 AUC _Initial_ was available for 28 patients

^b^ Bolinger 2000 AUC _Final_ was available for 27 patients

^c^ Wall 2009 AUC _Final_ was available for 22 patients

**Appendix 3 Raw data for safety (Table S2)**

**Table S2 Raw data for incidence of hepatotoxicity with different cutoff values.**

| **Reference** |  | **1350 µM × min** | | **1500 µM × min** | |
| --- | --- | --- | --- | --- | --- |
|  |  | **≤1350** | **＞1350** | **≤****1500** | **＞1500** |
| Okamoto 2014 | AUC(Initial) | 3/14 | 1/11 | 3/21 | 1/4 |
| Veal 2012 | AUC(mean) | 8/31 | 2/3 | NR | NR |
| Wall 2009 | AUC(Initial) | 3/22 | 1/2 | 3/22 | 1/2 |
| Wall 2009 | AUC(mean) | 2/20 | 2/4 | 4/24 | 0/0 |
| Wall 2009 | AUC(highest) | 2/19 | 2/5 | 3/21 | 1/3 |
| Michel 2011 | AUC(mean) | 4/42 | 3/15 | 5/56 | 2/11 |
| Vassal 2008 | AUC(mean) | 4/44 | 2/11 | 5/49 | 1/6 |
| Bouligand2003 | AUC(final) | 1/11 | 4/9 | 3/16 | 2/4 |
| Tran 2000 | AUC(Initial) | 0/15 | 1/10 | 0/16 | 1/9 |
| Tran 2000 | AUC(mean) | 0/16 | 1/9 | 0/18 | 1/7 |
| Tran 2000 | AUC(highest) | 0/7 | 1/18 | 0/12 | 1/13 |
| McCune2003 | AUC(mean) | 3/43 | 1/10 | NR | NR |
| Vassal 1996 | AUC(Initial) | NR | NR | 11/28 | 12/29 |

NR=not reported

**Appendix 4 Sensitivity analysis on each study’s affect on the summary estimates for efficacy (Table S3)**

**Table S3 Sensitivity analysis on each study’s affect on the summary estimates for efficacy (results were only reported when differing from primary analysis)**

| **Cutoff value** | **Rate of graft failure** | | |
| --- | --- | --- | --- |
|  | **Sensitivity analysis** | **No. of studies attributing data for sensitivity analysis** | **Primary**  **analysis** |
| 800 | - | 3 | 2.736[0.716, 10.453]  I^2^%=0 |
| 900 | - | 6 | 3.059[1.290, 7.258]  I^2^%=0 |
| 1000 | - | 4 | 1.787[0.564, 5.668]  I^2^%=0 |
| 1225 | - | 5 | 1.368[0.349, 5.361]  I^2^%=0 |

Note: -,=no meaningful difference

**Appendix 5 Sensitivity analysis on each study’s affect on the summary estimates for safety (Table S4)**

**Table S4 Sensitivity analysis on each study’s affect on the summary estimates for safety (results only reported when the conclusions differed from primary analysis)**

| **Cutoff value** | **Rate of VOD** | | |
| --- | --- | --- | --- |
|  | **Sensitivity analysis** | **No. of studies attributing data for sensitivity analysis** | **Primary**  **analysis** |
| 1350 | - | 7 | 0.370[0.205, 0.666]  I^2^%=0 |
| 1500 | 0.418[0.174,1.005] I^2^=0%^a^  0.427[0.159,1.148]  I^2^=0%^b^  0.443[0.191,1.026]  I^2^=0%^c^ | 5 | 0.409[0.182, 0.920]  I^2^%=0 |

Note: -,=no meaningful difference

^a^after removing study by wall;^b^after removing study by Bouligand;^c^after removing study by tran;

**Appendix 6 Quality appraisal of included studies (Table S5)**

**Table S5 Quality appraisal of included studies (indicators from Newcastle-Ottawa Scale of cohort studies)**

| **Reference** | **Outcome and cutoff value** | **Quality appraisal (indicators from New-Castle-Ottawa scale)** | | | | | | | | | | | | | | | | | **Total score** |
| --- | --- | --- | --- | --- | --- | --- | --- | --- | --- | --- | --- | --- | --- | --- | --- | --- | --- | --- | --- |
|  |  | 1 | 2 | | 3 | | 4 | | | 5A^a^ | | 5B^b^ | | 6 | | 7^c^ | | 8^d^ |  |
| Okamoto 2014 | graft failure, 800 µM × min | * | | * | | * | | * | * | | - | | * | | * | | * | | 8 |
| Okamoto 2014 | graft failure, 900 µM × min | * | | * | | * | | * | * | | - | | * | | * | | * | | 8 |
| Okamoto 2014 | graft failure, 1000 µM × min | * | | * | | * | | * | * | | - | | * | | * | | * | | 8 |
| Okamoto 2014 | graft failure, 1225 µM × min | * | | * | | * | | * | * | | - | | * | | * | | * | | 8 |
| Okamoto 2014 | VOD, 1350 µM × min | * | | * | | * | | * | * | | - | | * | | * | | * | | 8 |
| Okamoto 2014 | VOD, 1500 µM × min | * | | * | | * | | * | * | | - | | * | | * | | * | | 8 |
| Faraci 2017 | graft failure, 900 µM × min | * | | * | | * | | * | * | | - | | * | | * | | - | | 7 |
| Maheshwari 2014 | graft failure, 900 µM × min | * | | * | | * | | * | * | | - | | * | | * | | * | | 8 |
| Veal2012 | VOD, 1350 µM × min | * | | * | | * | | * | * | | * | | * | | * | | * | | 9 |
| Wall 2009 | graft failure, 800 µM × min | * | | * | | * | | * | * | | * | | * | | * | | * | | 9 |
| Wall 2009 | graft failure, 900 µM × min | * | | * | | * | | * | * | | * | | * | | * | | * | | 9 |
| Wall 2009 | graft failure, 1000 µM × min | * | | * | | * | | * | * | | * | | * | | * | | * | | 9 |
| Wall 2009 | graft failure, 1225 µM × min | * | | * | | * | | * | * | | * | | * | | * | | * | | 9 |
| Wall 2009 | VOD, 1350 µM × min | * | | * | | * | | * | * | | * | | * | | * | | * | | 9 |
| Wall 2009 | VOD, 1500 µM × min | * | | * | | * | | * | * | | * | | * | | * | | * | | 9 |
| Bolinger 2001 | graft failure, 800 µM × min | * | | * | | * | | * | * | | - | | - | | - | | * | | 6 |
| Bolinger 2001 | graft failure, 900 µM × min | * | | * | | * | | * | * | | - | | - | | - | | * | | 6 |
| Bolinger 2001 | graft failure, 1000 µM × min | * | | * | | * | | * | * | | - | | - | | - | | * | | 6 |
| Bolinger 2001 | graft failure, 1225 µM × min | * | | * | | * | | * | * | | - | | - | | - | | * | | 6 |
| Bolinger 2000 | graft failure, 800 µM × min | * | | * | | * | | * | * | | - | | - | | - | | * | | 6 |
| Bolinger 2000 | graft failure, 900 µM × min | * | | * | | * | | * | * | | - | | - | | - | | * | | 6 |
| Bolinger 2000 | graft failure, 1000 µM × min | * | | * | | * | | * | * | | - | | - | | - | | * | | 6 |
| Bolinger 2000 | graft failure, 1225 µM × min | * | | * | | * | | * | * | | - | | - | | - | | * | | 6 |
| Vassal 2008 | graft failure, 900 µM × min | * | | * | | * | | * | * | | * | | * | | - | | * | | 8 |
| Vassal 2008 | VOD, 1350 µM × min | * | | * | | * | | * | * | | * | | * | | - | | * | | 8 |
| Vassal 2008 | VOD, 1500 µM × min | * | | * | | * | | * | * | | * | | * | | - | | * | | 8 |
| Michel G 2011 | graft failure, 900 µM × min | * | | * | | * | | * | * | | * | | - | | * | | * | | 8 |
| Michel G 2011 | VOD, 1350 µM × min | * | | * | | * | | * | * | | * | | * | | * | | * | | 9 |
| Michel G 2011 | VOD, 1500 µM × min | * | | * | | * | | * | * | | * | | * | | * | | * | | 9 |
| McCune 2003 | graft failure, 900 µM × min | * | | * | | * | | * | * | | * | | * | | * | | * | | 9 |
| McCune 2003 | TRT≥grade 3, 1350 µM × min | * | | * | | * | | * | * | | * | | * | | * | | * | | 9 |
| Bouligand2003 | VOD, 1350 µM × min | * | | * | | * | | * | * | | * | | - | | - | | * | | 7 |
| Bouligand2003 | VOD, 1500 µM × min | * | | * | | * | | * | * | | * | | - | | - | | * | | 7 |
| tran2000 | VOD, 1350 µM × min | * | | * | | * | | * | * | | - | | - | | * | | * | | 7 |
| tran2000 | VOD, 1500 µM × min | * | | * | | * | | * | * | | - | | - | | * | | * | | 7 |
| Vassal 1996 | VOD, 1500 µM × min | * | | * | | * | | * | * | | - | | * | | - | | * | | 7 |

VOD=veno-occlusive disease; TRT, transplant-related toxicity

“*”, represents the score; “–”, the item(question) has no score; “/”, not applicable. NR= not reported

1. Indicates exposed cohort truly representative.

2. Non-exposed cohort drawn from a same source.

3. Ascertainment of exposure from a secure record.

4. Outcome of interest not present at start of study.

5A. Cohorts comparable on basis of main factor

5B. Cohorts comparable on other factors

6. Assessment of outcome of record linkage or independent blind assessment.

7. Follow-up long enough for outcomes to occur.

8. Complete accounting for cohorts

**Appendix 7 Forest plot for different outcomes at each cutoff value (Figure S1-S12)**

**Appendix 7.1 Forest plot for rate of** **graft failure at each cutoff value (Figure S1-S8)**

**
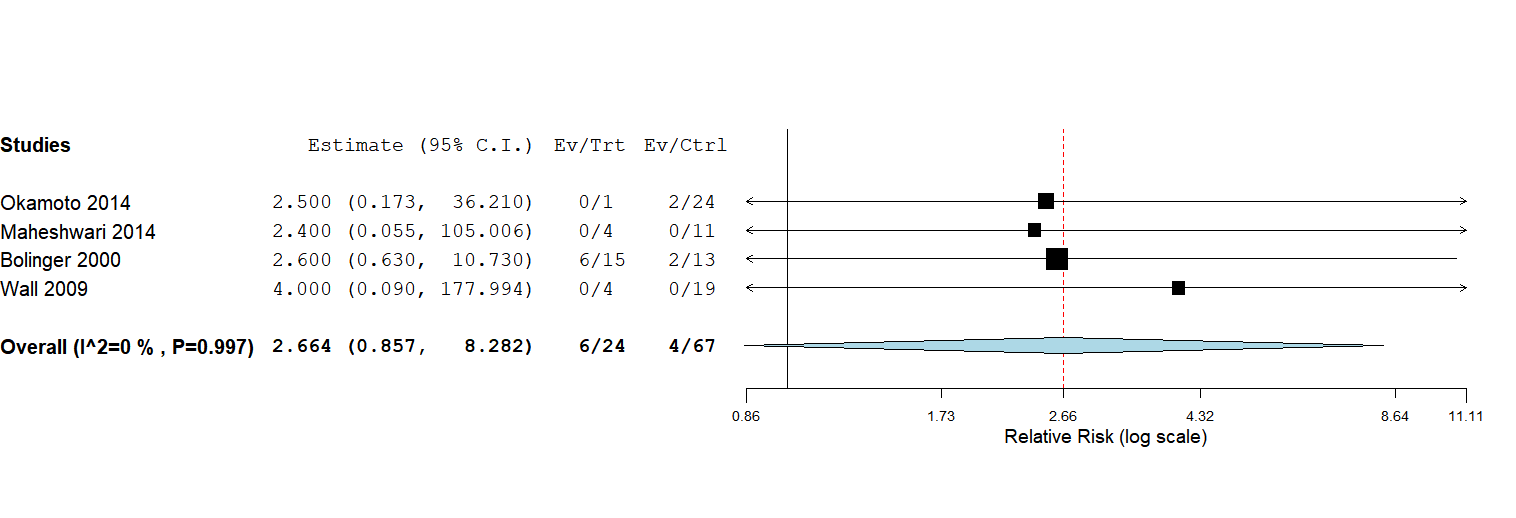
****Figure S1 Meta-analysis for the incidence of graft failure (****first dose AUC of <800 µM × min comparison with ≥800 µM × min, RR <1 favors ≥800 µM × min)**

**
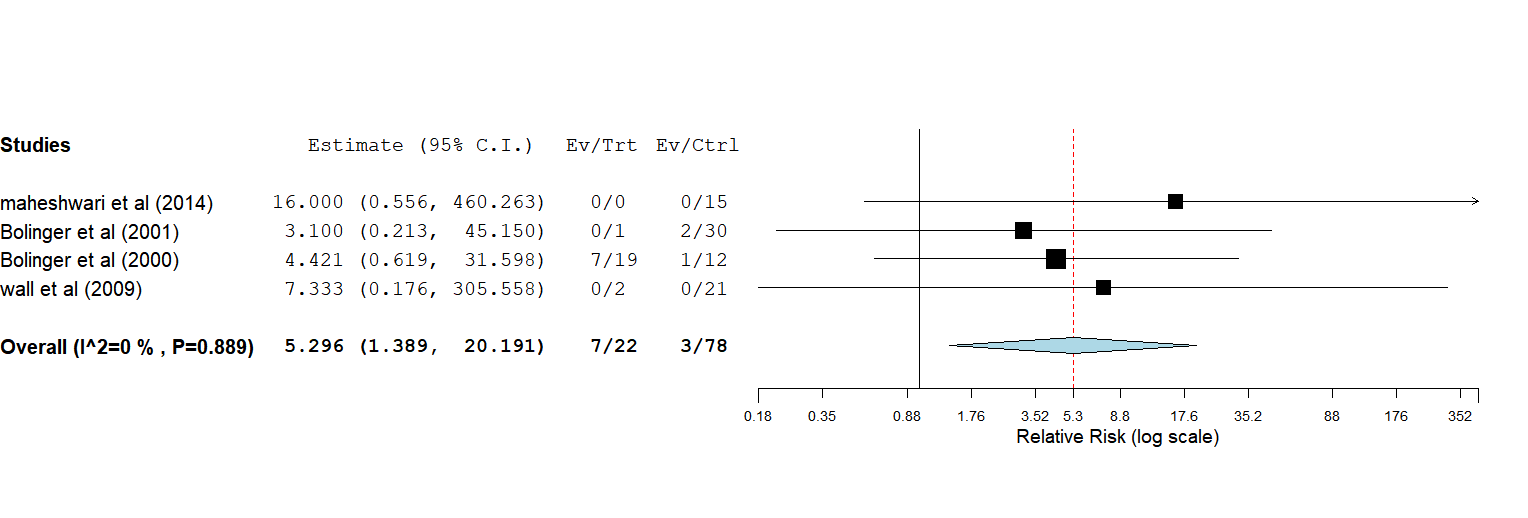
**

**Figure S2 Meta-analysis for the incidence of graft failure (mean AUC of <800 µM × min comparison with ≥800 µM × min , RR <1 favors ≥800 µM × min)**

**
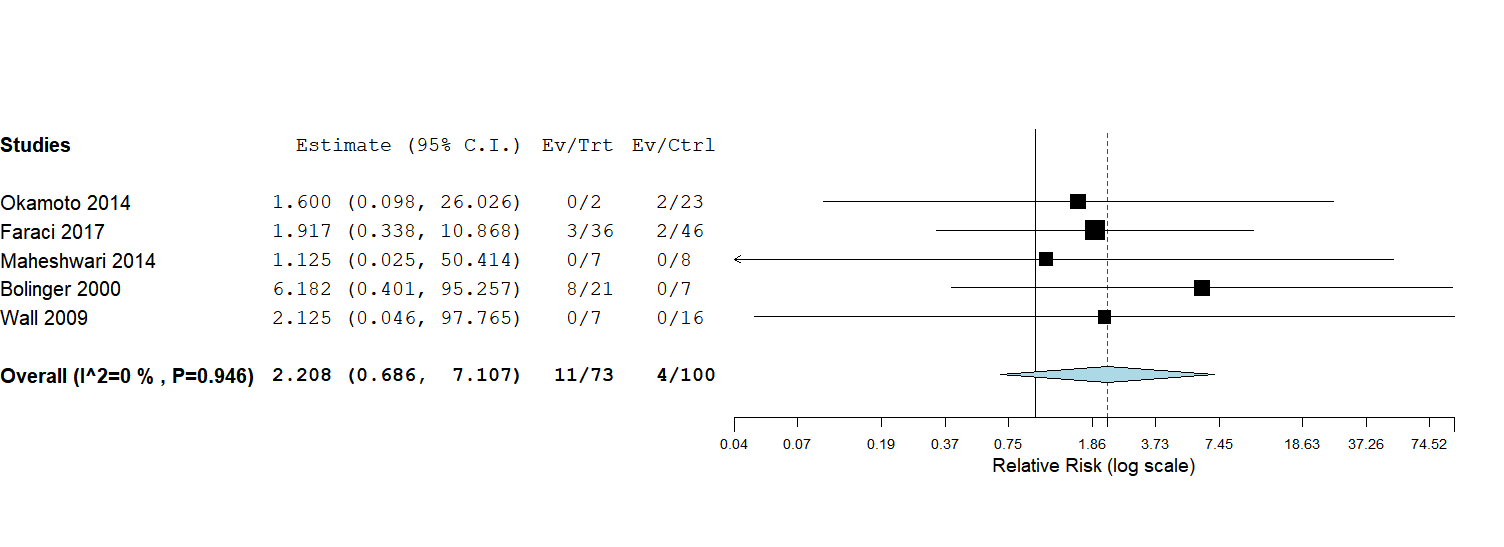
****Figure S3 Meta-analysis for the incidence of graft failure** **(****first dose AUC of** **<****900 µM × min comparison with** **≥900 µM × min , RR <1 favors ≥ 900 µM × min)**

**
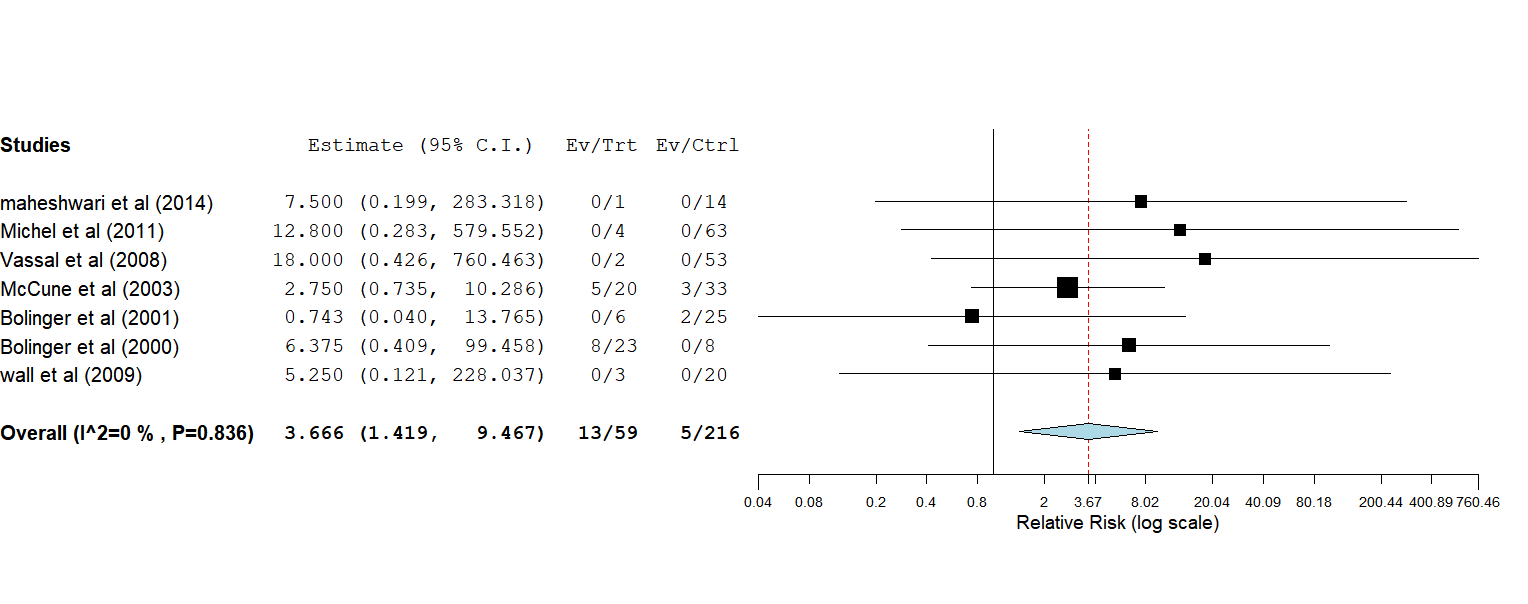
****Figure S4 Meta-analysis for the incidence of graft failure (mean AUC of <900 µM × min comparison with ≥ 900 µM × min , RR <1 favors ≥ 900 µM × min)**

**
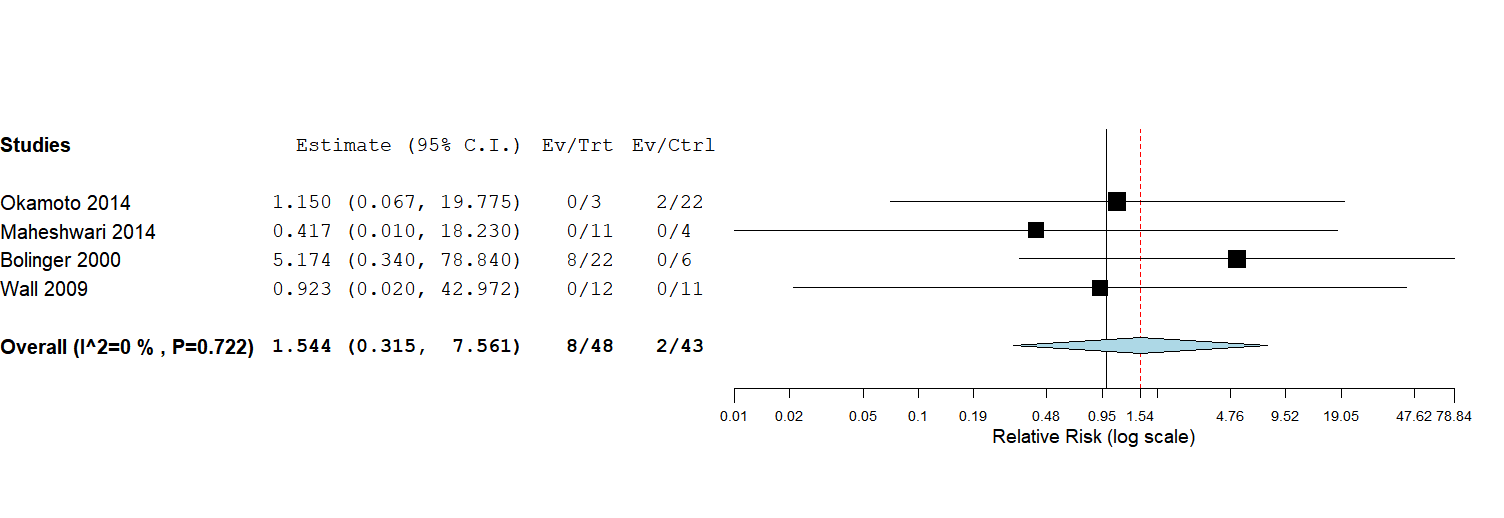
****Figure S5 Meta-analysis for the incidence of graft failure (** **first dose AUC of <1000 µM × min comparison with ≥1000 µM·ּּmin , RR <1 favors ≥1000** **µM × min).**

**
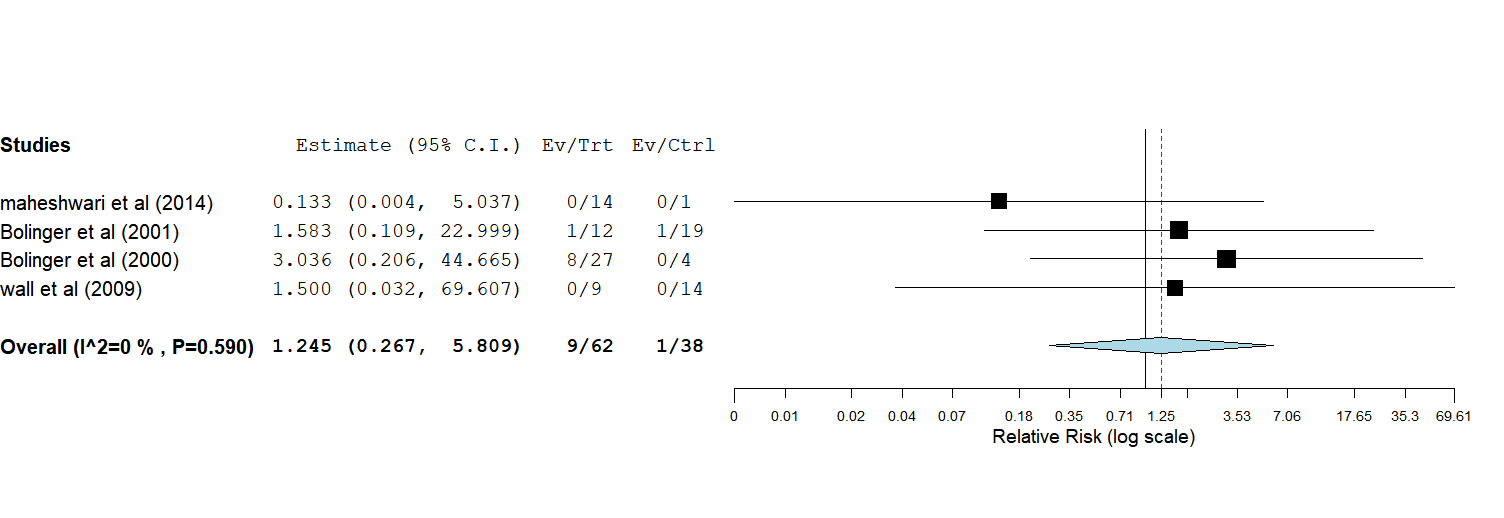
Figure S6 Meta-analysis for the incidence of graft failure ( mean AUC of <1000 µM × min comparison with ≥1000 µM × min , RR <1 favors ≥1000 µM × min).**


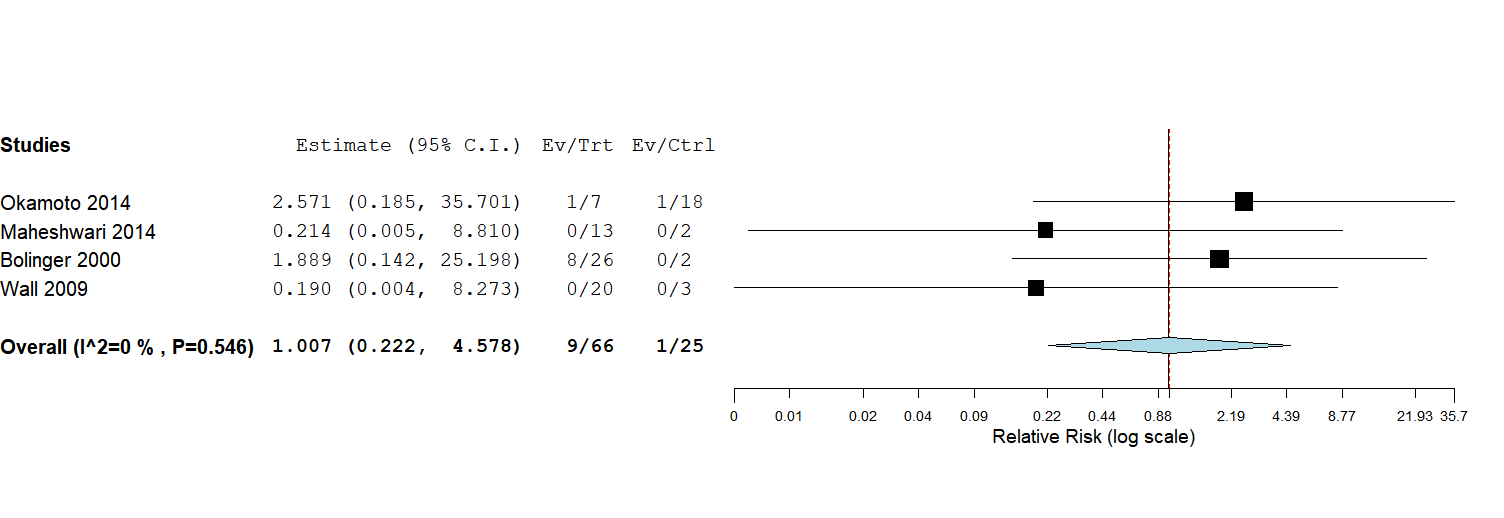
**F****igure S7 Meta-analysis for** **the incidence of graft failure (first dose AUC of <1225 µM × min comparison with ≥1225 µM × min, RR <1 favors ≥1250 µM × min)**


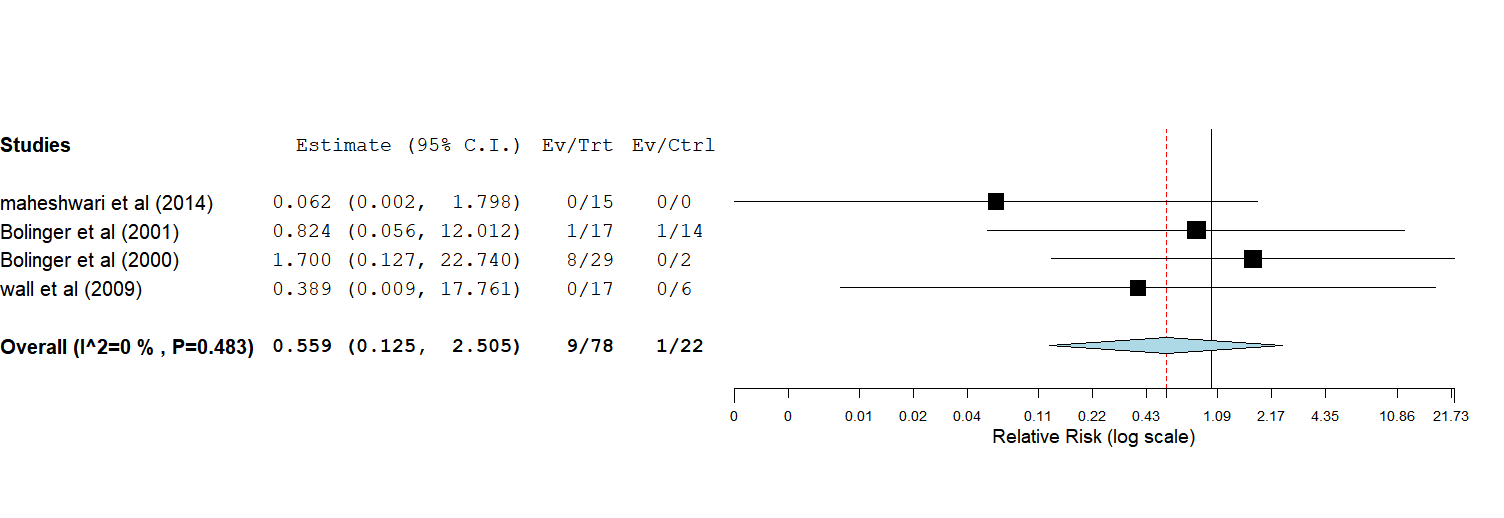


**Figure S8 Meta-analysis for the incidence of graft failure (mean AUC of <1225 µM × min comparison with ≥1225 µM × min, RR <1 favors ≥1250 µM × min )**

**Appendix 7.2 Forest plot for subgroup analysis of** **rate of graft failure at each cutoff value (Figure S9-S12)**

**
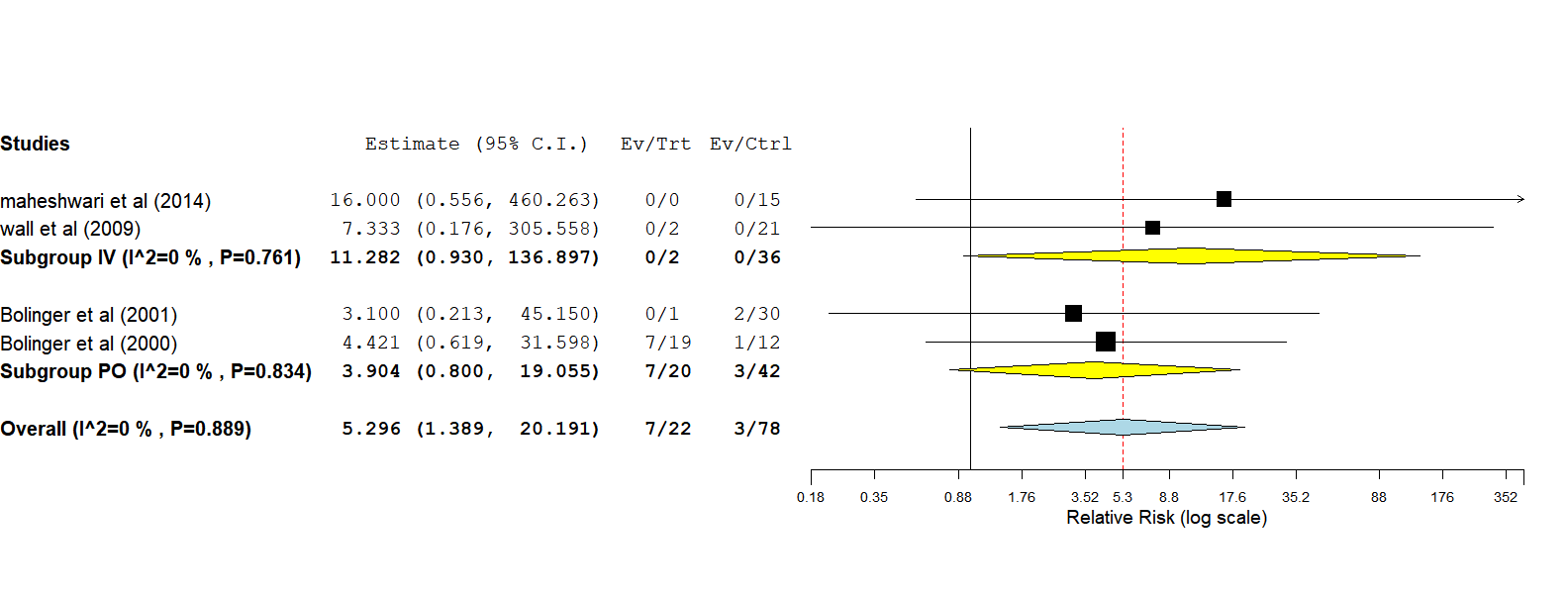
**

**Figure S9 Subgroup analysis for** **the incidence of graft failure stratified by administration route (mean AUC of <800 µM × min comparison with ≥800 µM × min, RR <1 favors ≥800 µM ×min)**


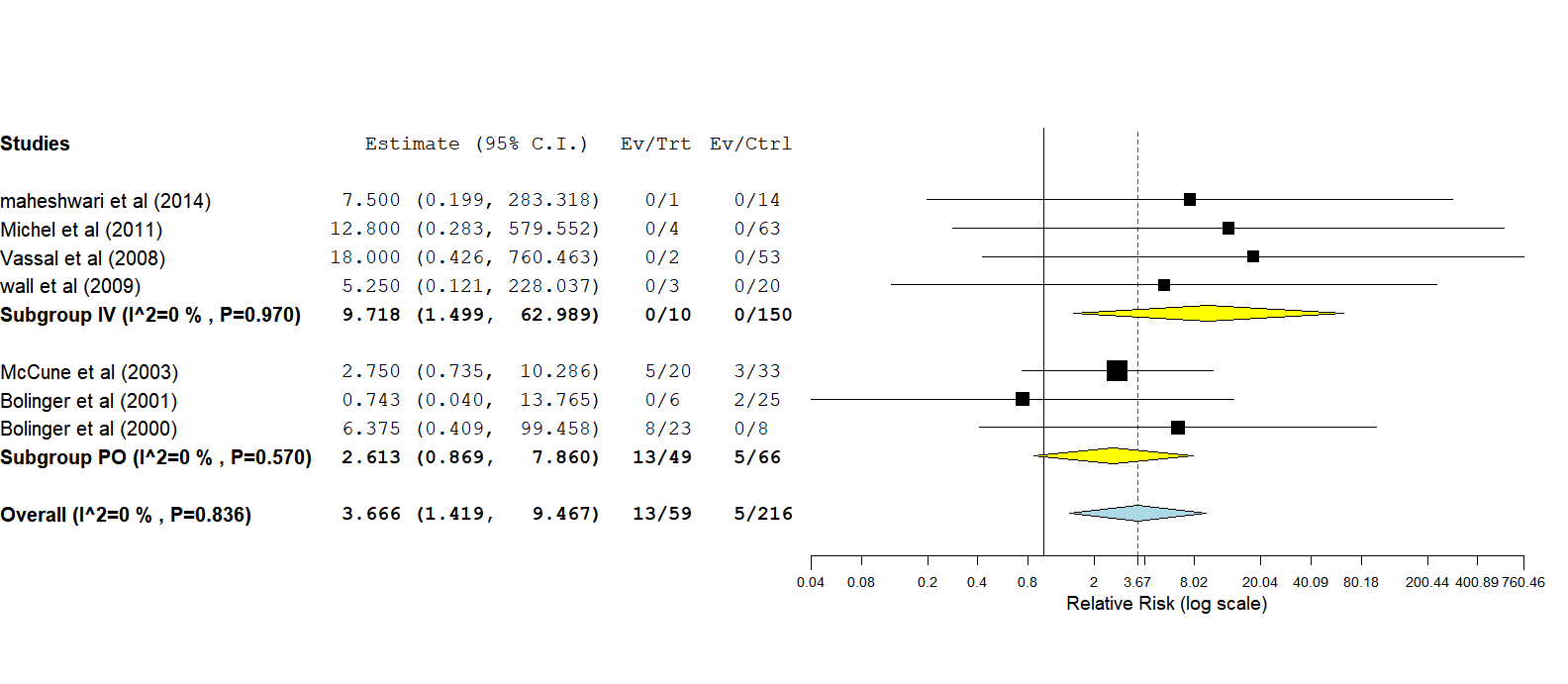


**Figure S10 Subgroup analysis for the incidence of graft failure stratified by administration route (mean AUC of <900 µM × min comparison with ≥900 µM × min, RR <1 favors ≥900 µM ×min)**

**
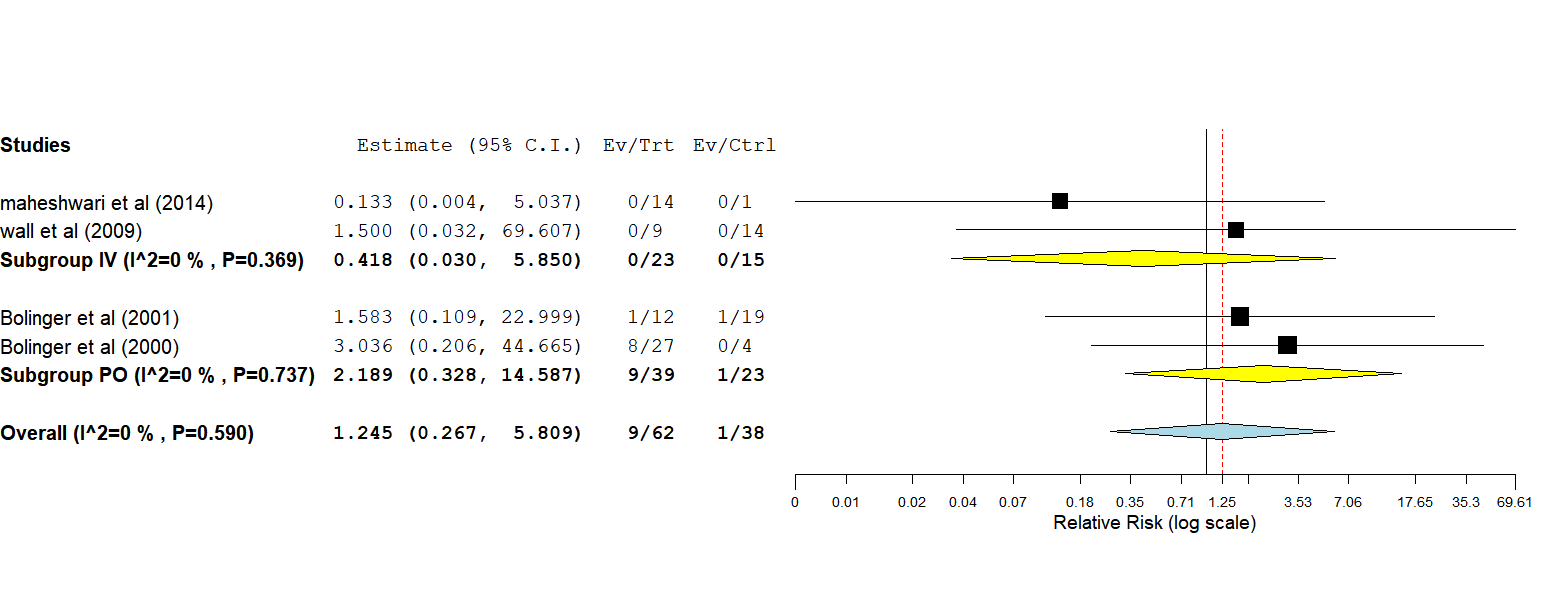
**

**Figure S11 Subgroup analysis for the incidence of graft failure stratified by administration route (mean AUC of <1000 µM × min comparison with ≥1000 µM × min, RR <1 favors ≥1000 µM ×min)**

**
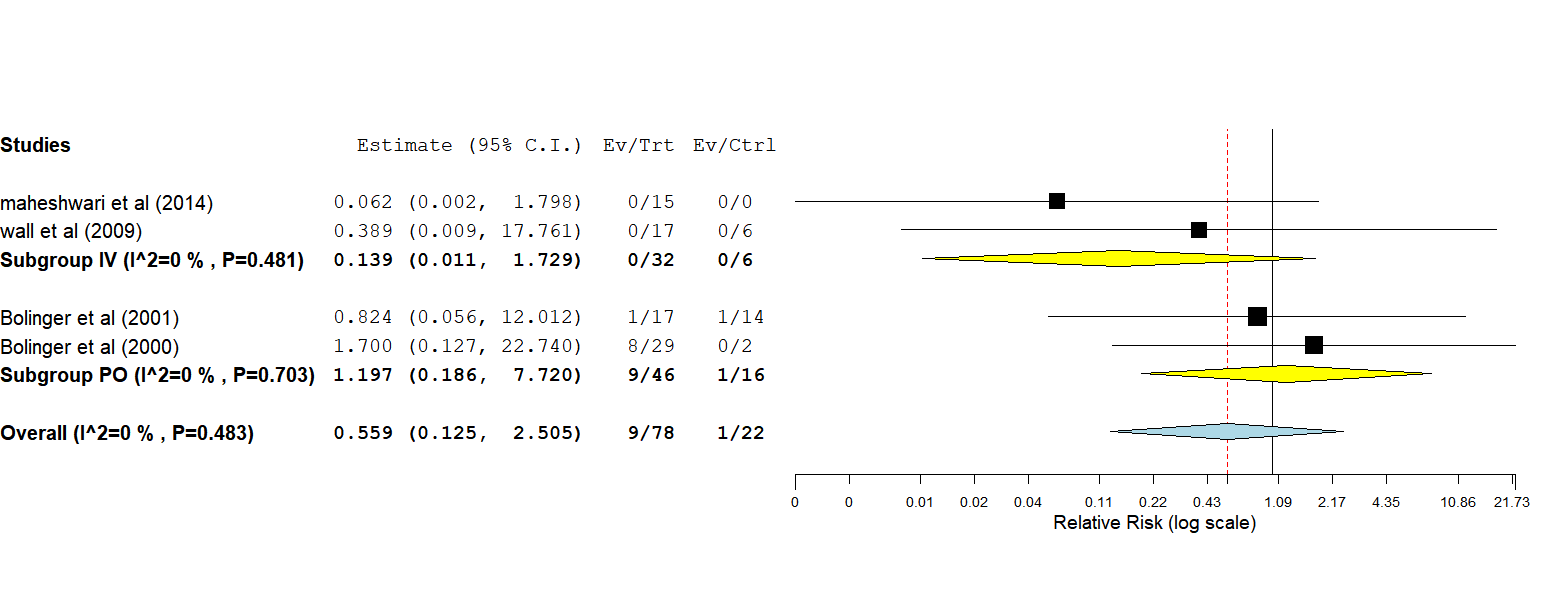
**

**Figure S12 Subgroup analysis for the incidence of graft failure stratified by administration route(mean AUC of <1225 µM × min comparison with ≥1225 µM × min, RR <1 favors ≥1225 µM ×min)**

**Appendix 7.3** **Forest plot for safety at each cutoff value (Figure S13-S16)**

**
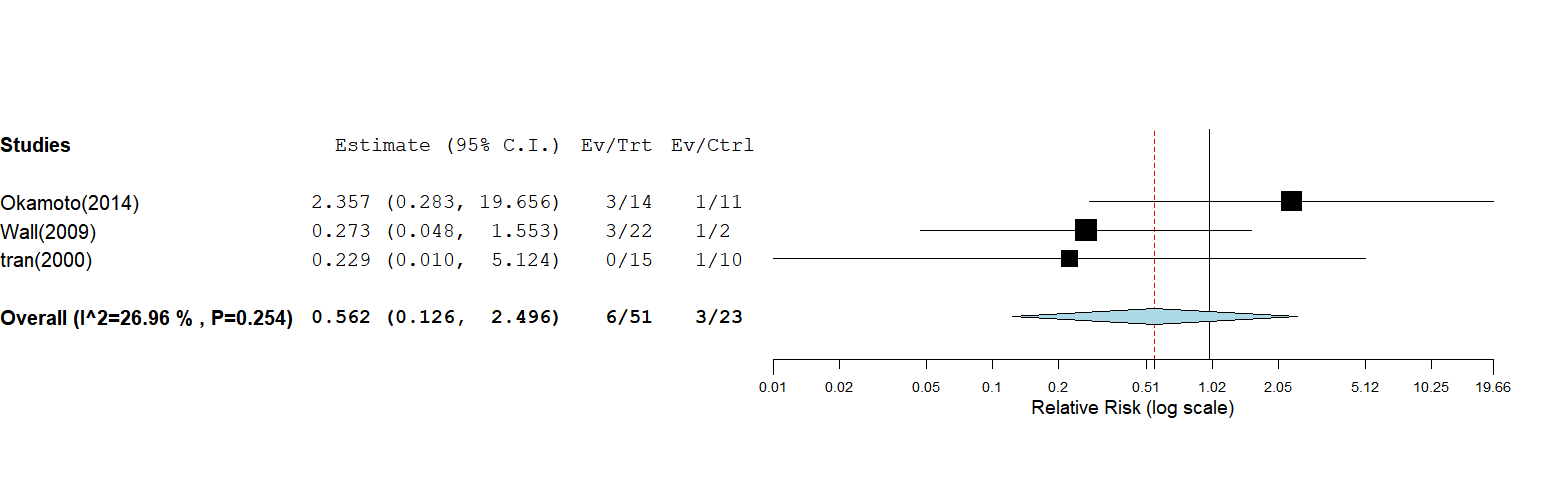
**

**Figure S13 Meta-analysis for incidence of VOD or transplant-related toxicity (liver≥grade 3) ( first dose AUC of <1350 µM × min comparison with ≥1350 µM × min , RR <1 favors ≥1350 µM × min)**

**
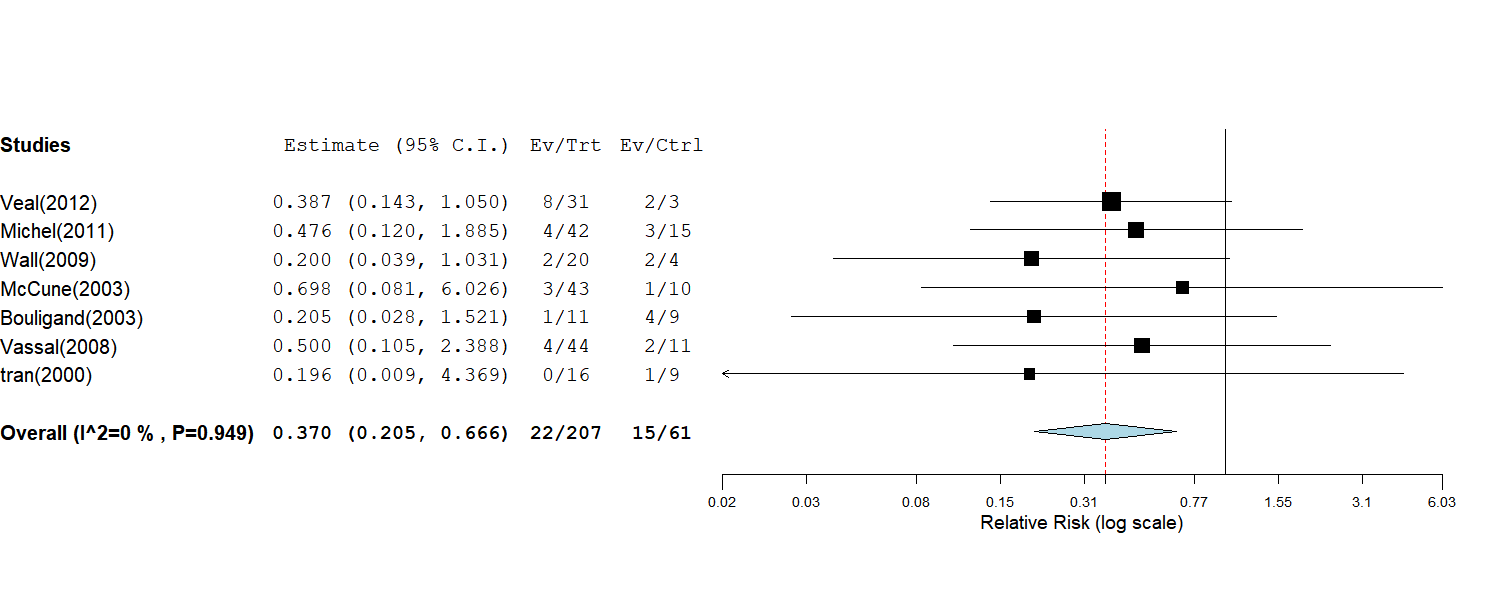
****Figure S14 Meta-analysis for incidence of VOD or** **transplant-related toxicity (liver≥grade 3) (mean AUC of <1350 µM × min comparison with ≥1350 µM × min , RR <1 favors ≥1350 µMmin)**

**
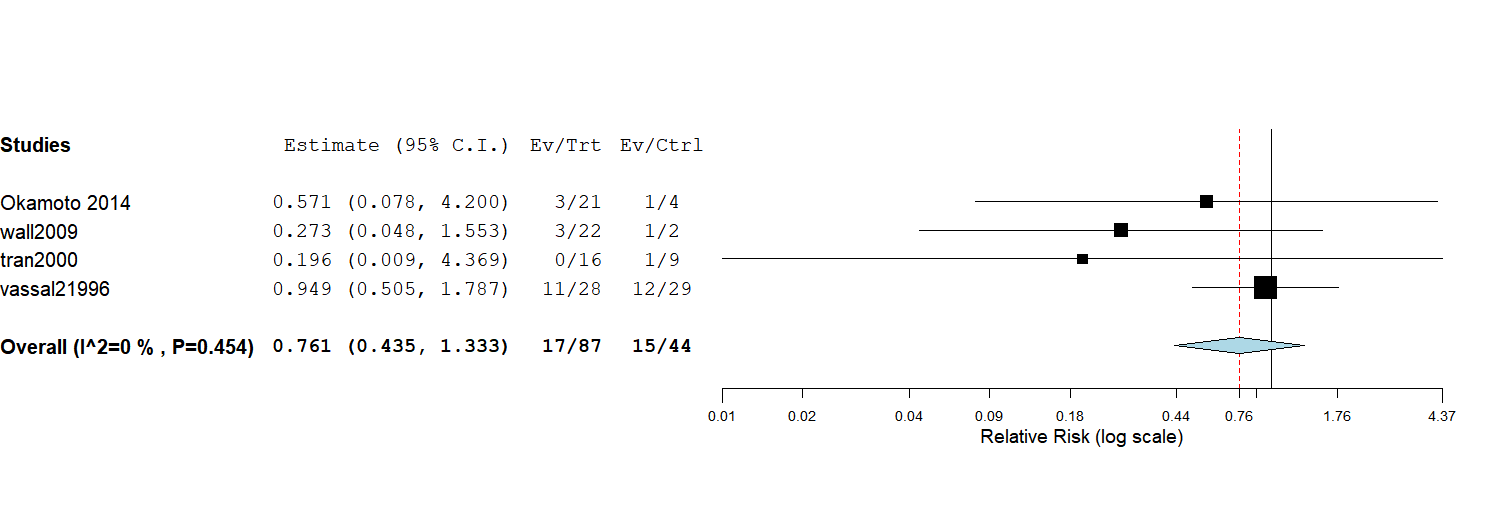
**

**Figure S15 Meta-analysis for incidence of** **VOD or** **transplant-related toxicity (liver≥grade 3) (first dose AUC of <1500 µM*min /L comparison with ≥1500 µM × min , RR <1 favors ≥1500 µM × min)**

**
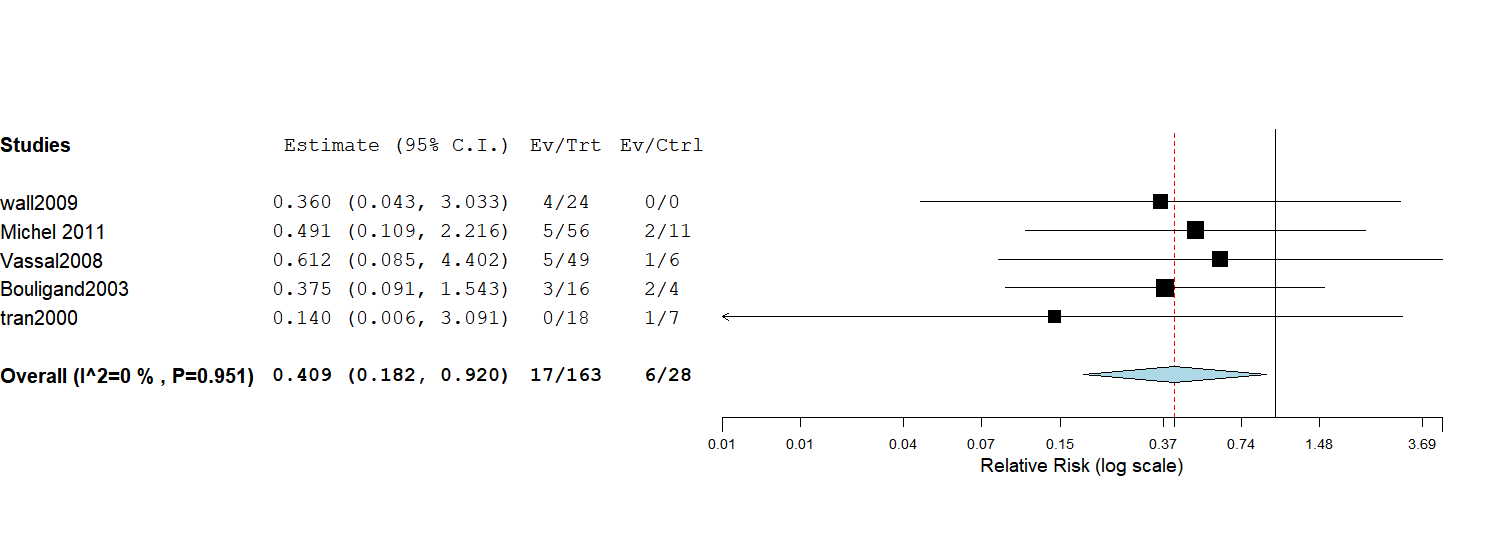
**

**Figure S16 Meta-analysis for incidence of VOD or transplant-related toxicity (liver≥grade 3) (mean AUC of <1500 µM × min comparison with ≥1500 µM × min, RR <1 favors ≥1500 µM × min)**

**Appendix 7.4 Forest plot for subgroup analysis of rate of veno-occlusive disease (VOD) or transplant-related toxicity (liver≥grade 3) at each cutoff value (Figure S17-S20)**

**
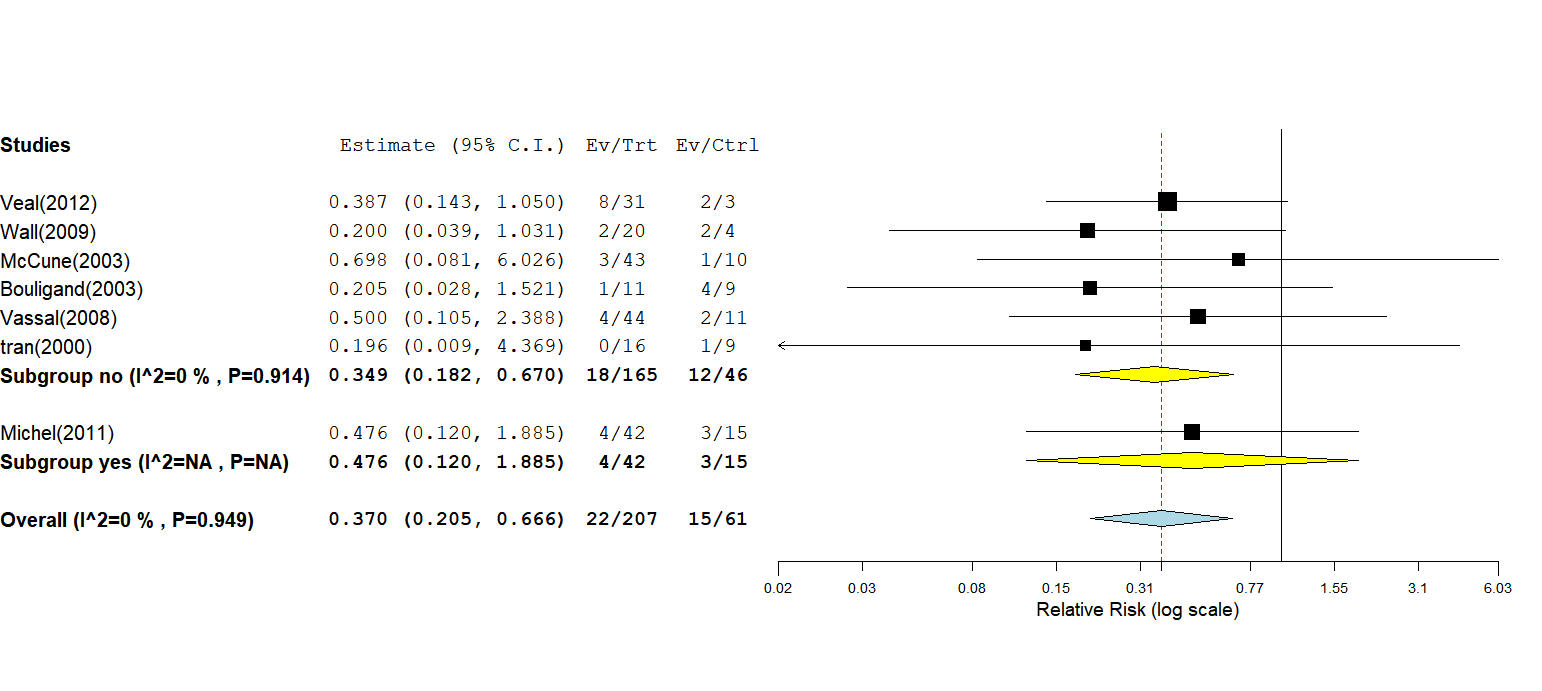
**

**Figure S17 Subgroup analysis for rate VOD or transplant-related toxicity (liver≥grade 3) stratified by with VOD prophylaxis therapy or not****(mean AUC of <1350 µM × min comparison with ≥1350 µM × min, RR <1 favors ≥1350 µM × min)**

**
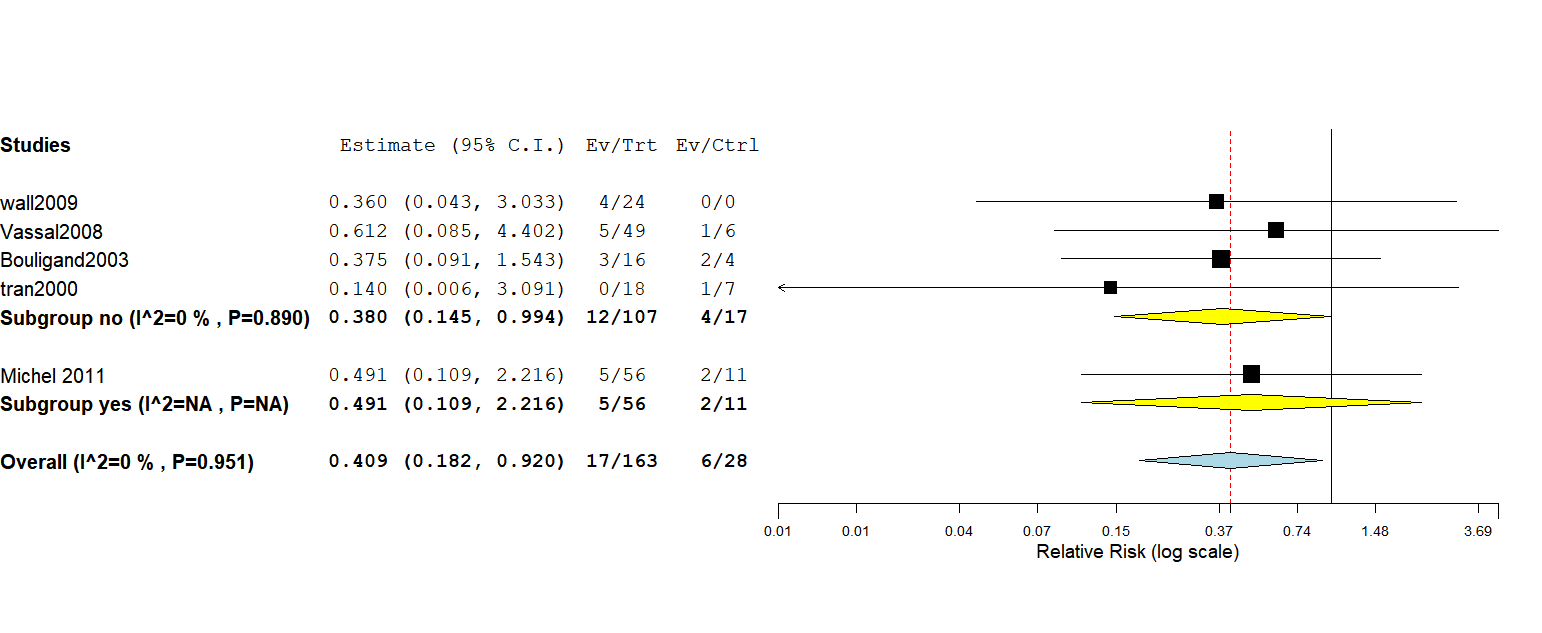
**

**Figure S18 Subgroup analysis for rate VOD or transplant-related toxicity (liver≥grade 3) stratified by with VOD prophylaxis therapy or not(mean AUC of <1500 µM × min comparison with ≥1500 µM × min, RR <1 favors ≥1500 µM × min)**

**
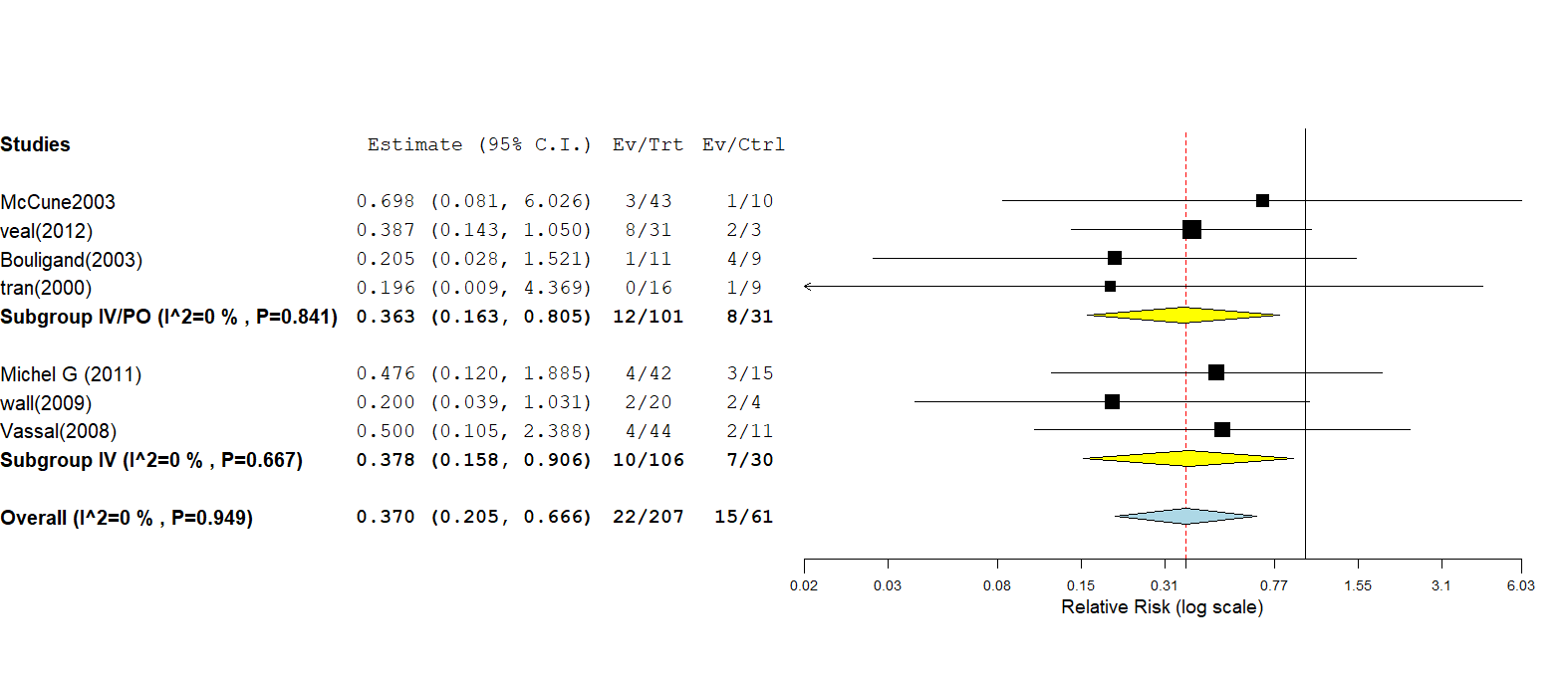
**

**Figure S19 Subgroup analysis for rate VOD or transplant-related toxicity (liver≥grade 3) stratified by** **administration route(mean AUC of <1350 µM × min comparison with ≥1350 µM × min, RR <1 favors ≥1350 µM ×min)**

**
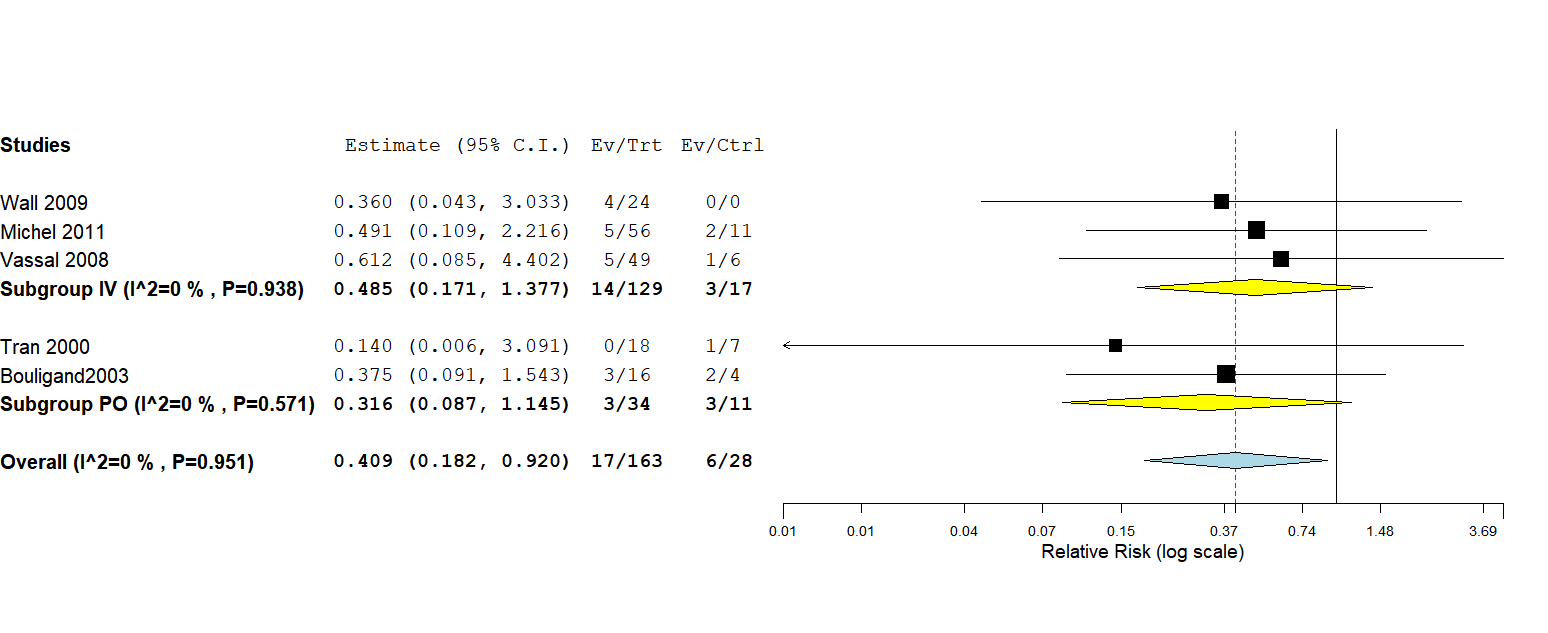
**

**Figure S20 Subgroup analysis for rate VOD or transplant-related toxicity (liver≥grade 3) stratified by** **administration route (mean AUC of <1500 µM × min comparison with ≥1500 µM × min, RR <1 favors ≥1500 µM × min)**
